# A Full-Body IMU-Based Motion Dataset of Daily Tasks by Older and Younger Adults

**DOI:** 10.1038/s41597-025-04818-y

**Published:** 2025-03-29

**Authors:** Loreen Pogrzeba, Evelyn Muschter, Simon Hanisch, Veronica Y. P. Wardhani, Thorsten Strufe, Frank H. P. Fitzek, Shu-Chen Li

**Affiliations:** 1 Technische Universität Dresden, Centre for Tactile Internet with Human-in-the-Loop, 01062 Dresden, Germany; 2 Technische Universität Dresden, Research Hub 6G-life, 01062 Dresden, Germany; 3 Technische Universität Dresden, Chair of Lifespan Developmental Neuroscience, 01062 Dresden, Germany; 4 Karlsruhe Institute of Technology, Chair of Privacy and Security, 76131 Karlsruhe, Germany; 5 Technische Universität Dresden, Deutsche Telekom Chair of Communication Networks, 01062 Dresden, Germany

**Keywords:** Ageing, Skeleton, Biomedical engineering

## Abstract

This dataset (named *CeTI-Age-Kinematics*) fills the gap in existing motion capture (MoCap) data by recording kinematics of full-body movements during daily tasks in an age-comparative sample with 32 participants in two groups: older adults (66–75 years) and younger adults (19–28 years). The data were recorded using sensor suits and gloves with inertial measurement units (IMUs). The dataset features 30 common elemental daily tasks that are grouped into nine categories, including simulated interactions with imaginary objects. Kinematic data were recorded under well-controlled conditions, with repetitions and well-documented task procedures and variations. It also entails anthropometric body measurements and spatial measurements of the experimental setups to enhance the interpretation of IMU MoCap data in relation to body characteristics and situational surroundings. This dataset can contribute to advancing machine learning, virtual reality, and medical applications by enabling detailed analyses and modeling of naturalistic motions and their variability across a wide age range. Such technologies are essential for developing adaptive systems for applications in tele-diagnostics, rehabilitation, and robotic motion planning that aim to serve broad populations.

## Background & Summary

Movements are essential for humans to interact with environments. Motor abilities are crucial for the elderly to maintain autonomy^1^ and independently perform daily tasks^2^. Neurocognitive research shows that physical activities improve brain functions serving memory or attention, even in old age^3–5^. Applied research makes use of movement analyses to assess physical and cognitive declines in the elderly and the progressions of neurodegenerative disorders in patients^6–10^. The typical, non-pathological process of aging entails structural changes in the brain (e.g., shrinkage of brain volumes) and body (e.g., reduced range of motion in body joints)^11–14^. These changes lead to limited capabilities for motion planning and execution, resulting in either increased movement fluctuations^15^ or reduced motion flexibility, as reflected in problematic gait patterns^16^. Moreover, individuals differ in their kinematic variability which, in part, are associated with individual differences in neural variability^17^. These findings underscore the need for representative data on full-body movements of younger and older people performing daily tasks to assess and understand age-related differences in motion characteristics. Advanced applications of real-time movement analysis and modeling can benefit from machine learning (ML) approaches that are trained with rich data of daily movements from demographically representative samples.

To the end, the *CeTI-Age-Kinematics* dataset includes two age groups—older (66–75 years) and younger (19–28 years) adults—to provide data covering a broader age range. Recent sensor technologies make the acquisitions of kinematic MoCap data more approachable to foster movement research for applications in tele-diagnostics^18^, human digital twins for medical (remote) assessment^19,20^ and exergaming^21–23^. However, integrating diverse populations into motion datasets remains a challenge^24^. Existing datasets based on various sensors typically focus on young- or middle-aged adults executing specific movement categories, e.g., selected daily tasks^25–28^, postural balance^29,30^, exercises for physical therapy^31^, or walking for gait analyses^32–37^. Complementing existing datasets, this dataset uniquely focuses on capturing full-body, including hand, movements from 30 common daily tasks in nine categories (e.g., targeted reaching, balance control, object transfer, and other object interactions) with repetitions that were performed also by older people beyond retirement age besides younger adults. By including a sex-stratified, healthy sample of elderly participants, this dataset allows for investigations into how aging may influence sensorimotor capabilities in daily tasks. Understanding potential age differences in movement variability is crucial for developing adaptive systems, because such information supports the design for individualized interventions and algorithms for robust action recognition and segmentation^38–41^. By also including older adults’ motion patterns, this dataset promotes age-inclusivity^42,43^, enabling researchers to consider age-related functional changes^44^ in system and technical designs to better address elderly needs^45^. Moreover, previous datasets often lacked sufficient repetitions of the same movements^41^ and did not provide adequate details about the environments in which daily movements were performed by older adults^46,47^. The omission of such information is significant, as spatial conditions can influence movement execution^48–50^. Laboratory studies, although being well controlled, may not fully represent real-word behaviours. Our dataset improves upon these aspects by providing multiple task repetitions executed individually by the participants using well-controlled and documented task variations of the execution speed, object involvement, and spatial conditions. This dataset was carefully designed to capture a wide range of naturalistic movements performed by participants in individually customized setups (i.e., unrestricted movements in all anatomical planes, movement execution in comfortable standing positions and reaching distances) to reflect personal anthropometric limitations, preferences, and particularities in the MoCap data. Besides actual physical objects, we also included imaginary objects to simulate interaction scenarios similar to those in virtual reality, where objects are typically not physically present or only visually represented.

In summary, the *CeTI-Age-Kinematics* dataset includes a rich variety of data contents and contexts to capture the complexity of daily full-body motions in the elderly as well as in young adults. It provides a comprehensive and realistic representation of elemental daily movements. The age-related differences in movement characteristics may have implications for the methods used for motion analysis and modeling. This dataset holds utilities for domains such as motion planning for robots^51,52^, intention sensing^10^, semantic action recognition based on motion in combination with instructions^53^, person identification either with the purpose of designing personalized systems^41,54^, biometric recognition^55,56^, or anonymization^57^. The data can be further explored in use cases of neurorehabilitation^58^ and ergonomics^59^.

## Methods

### Participants

Data was collected from 32 participants (17 female/15 male, mean age = 52.0 ± 23.5 years, 2 left-handed) in two age groups, covering older adults (OA) aged 66–75 years and younger adults (YA) aged 19–28 years (see Table 1 for details on group-specific sample size, selected demographic and basic anthropometric variables with descriptive statistics). The study was approved by the Ethical Committee of the Technische Universität Dresden (approval number: SR-EK-5012021) and was conducted in accordance with the Declaration of Helsinki^60^. All participants voluntarily signed an informed consent form and provided the permission for the use and publication of the pseudonymized data. A unique identifier was assigned to each participant to ensure data pseudonymization. The questionnaire data was gathered and administered through Research Electronic Data Capture (REDCap)^61,62^ that is hosted at Technische Universität Dresden.

**Table 1:** Selected key demographic data of the *CeTI-Age-Kinematics* sample.

	Younger adults (YA)	Older adults (OA)
Age range (years)	19–28	66–75
N	12	20
Sex (*N*_*female*_/*N*_*male*_)	7 / 5	10 / 10
Age mean ± SD (years)	22.3 (3.0)	69.8 (2.7)
Height mean ± SD (cm)	173.3 (8.2)	167.8 (7.2)
Mass mean ± SD (kg)	66.8 (11.4)	74.5 (14.0)
Handedness (*N*_*right*_/*N*_*left*_)	12 / 0	18 / 2
Reported impairments (N)	0	5

Shown here are group sums or averages, whereas individual participant data are listed in the Supplementary Table S1. Mean values for age, body mass, and height were calculated over all participants (see columns age, mass, and a01 in Table S1). An overview of all demographic information collected is provided in the Supplementary Table S5.

All participants self-reported having normal or corrected-to-normal vision and hearing, along with the ability to perform autonomous locomotion without the need for mobility aids. Demographic questions were asked to gather information about the participants’ educational background, physical activities, as well as their clothing and shoe sizes. The individual history of musculoskeletal pain, if applicable, was documented using the Nordic Musculoskeletal Questionnaire (NMQ)^63^ in its German version provided by the Federal Institute for Occupational Safety and Health [^64^, p. 87–88], in addition to relevant motion impairments potentially affecting the ability to perform daily movements (see Reported impairments in Table 1 and other details in Supplementary Information, Section 1). Moreover, the Edinburgh Handedness Inventory (EHI)^65^ was employed to assess the handedness score for each participant. This comprehensive approach was taken to provide a thorough assessment of participants’ health status, fitness level, body composition, and their potential impacts on the participants’ capacity to perform motions effectively and consistently. Anthropometric data were systematically collected, detailing both upper and lower limbs (for details of these data see Supplementary Information, Table S2). Other details about the contents of the questionnaires and assessed variables can be found in the Supplementary Information (see Table S5) and in the files named participants.tsv and participants.json (see Data Records).

### Wearable IMU-based Motion Capture (MoCap)

**Fig. 1:**
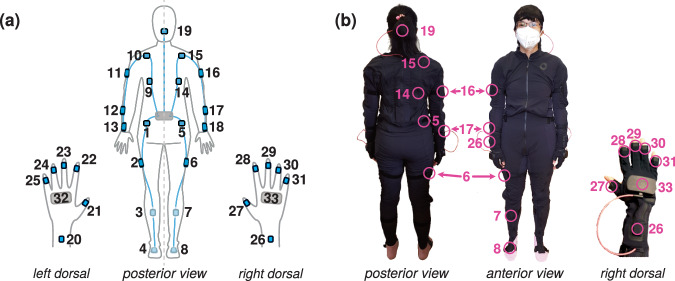
Distribution and locations of the MoCap sensors on the ultilized sensor suit and gloves. (**a**) Schematic visualization of sensor distribution on the body. (**b**) Sensor location on the full-body textile suit and data glove (specified for right body side).

Kinematic data was recorded at 100 Hz using a full-body wearable data suit (Smartsuit Pro Version 1) and data gloves (Smartgloves^66^), manufactured by Rokoko Electronics, Denmark (see Fig. 1b).

The data suit incorporates a total of 19 pre-installed IMU sensors that are seamlessly integrated into a thin, soft textile suit (see Fig. 1a for a schematic visualization) so that they can be easily removed for maintenance purposes (incl. cleaning of the textile part of the suit). The data suits are available in four sizes (S, M, L, XL) that come with Velcro straps for securing good fit to ensure stable sensor positioning during movements. The placements of the sensors are designed to capture comprehensive body movements, with one sensor dedicated to tracking head movements and six sensors allocated per limb (three per each arm and leg), and six for the torso. Together they allow precise recordings of head, limb, and spine motions. Specifically, all sensors of the suit are based on 9-degrees of freedom IMUs and incorporate hardware-synchronized signals of both a 16-bit tri-axial accelerometer and gyroscope (Bosch Sensortec, BMI160; see^67^ for other technical details about the sensors), along with an external magnetometer. The built-in IMUs enable on-chip motion-based action/gesture recognition, such as step detection^67^. The accelerometer and gyroscope measure linear acceleration and angular velocity, respectively^68^. The external magnetometer provides the absolute direction of the earth’s local magnetic field and can be utilized as a reference for the IMU’s absolute heading to improve the measurement accuracy through sensor fusion (see^67,69^ for technical details). Furthermore, the data suit uses a static pose to align each sensor’s geometrical axes with the orientation of body segments^70^, requiring an accurate assessment of the calibration pose as a reference (see the section on movement tasks c01 below for further details).

The data gloves are designed without built-in magnetometers to enhance resistance to magnetic interference^66^. For precise motion tracking of the fingers and wrist, each data glove features seven sensors that are located at the wrist and the distal phalanges of the fingers and thumb (see inset of right hand in dorsal view in Fig. 1b). The data gloves provide a 3-dimensional (3d) orientation accuracy of  ± 1 degree with a latency of  ± 20 ms^66^.

Together the data suit and gloves are quite commonly utilized for live MoCap streaming, specifically for character animation and virtual production that have latency requirements in the range of 20-30 ms^71^ to ensure responsive avatar movements in real-time. In past biomechanical studies, the measurement accuracy of such an IMU-based data suit in comparison to optoelectronic^72,73^ and video-based^74^ MoCap systems has been demonstrated as sufficient. For controlled hip movements (e.g., flexion/extension), the data suit showed a high agreement with an OptiTrack MoCap system (Corvallis, USA), with a root mean square error (RMSE) of 1.38 − 1.81° and Pearson correlation coefficients *r *≥ 0.98^72^. In terms of lower limb movements, the data suit also exhibited high agreements with the Qualisys MoCap system (Göteborg, Sweden) for hip and knee joints (RMSE  < 6°, *r *≥ 0.98) but a slightly weaker agreement for ankle dorsiflexion-plantarflexion (RMSE = 5. 1°, *r* = 0.80)^73^.

### Movement tasks

**Fig. 2:**
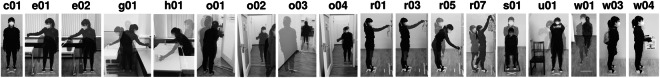
Illustrated motion sequences of selected movement tasks, with single letters indicating task categories, numbers denoting task variations (cf. Table 2 for details): c for *calibration* and neutral pose; e for *elevating* tool cases; g for reach-*grasp*-relocate; h for reach-grasp-*handover*-relocate; o for *opening a door*; r for *reaching*, placing, and *retrieving* an object from a hook; s for 5-repetition *sit-to-stand* test; u for *upper limb* task; and w for *walking* tasks.

The dataset incorporates 30 common daily movement tasks that were performed in naturalistic and unrestricted settings to account for variations in speed, style, and environmental conditions (see Fig. 2 and Table 2). The selection and design of these movement tasks follow several key principles as detailed in the Supplementary Information (Section 2). In the dataset these movement tasks are labeled by single letters with numbers behind the letters indicating task variations (see Fig. 2, cf. Table 2): specifically, c for *calibration* and neutral pose; e for *elevating* toolcases; g for a reach-*grasp*-relocate action; h for a reach-grasp-*handover*-relocate action; o for actions involving *opening a door*; r for *reaching for*, placing, and *retrieving* an object from a hook; s for a 5-repetition *sit-to-stand* test (5RSTST); u for an *upper limb* task; and w for *walking* tasks. Each movement was executed in 5 repetitions (except for c01 and u01 which were only performed twice). The speed of execution was self-paced for all tasks, except for the task 5RSTST, which required participants to perform as quickly as possible^75^. The self-paced speed encompassed both comfortable moderate pace and a faster pace that simulated situations of being in a hurry (see Table 2, o03–06, w01–04). Before executing each movement task, the participants were verbally instructed with the task descriptions. For tasks involving interactions with objects, the spatial setups were tailored to each individual participant. For example, object distances (d) were adjusted according to participant-defined (p-def.) preferences, as stated in Table 2 and detailed in the Supplementary Information (Section 3, cf. Fig. S1). All movement recording details are documented in the session protocols, which are available in the Data Descriptor (see Data Records). Additionally, the dataset also includes exemplary video sequences of the recorded tasks. In the following, the tasks are summarized by categories.

**Table 2:** Overview of movement tasks (sorted alphabetically by the task label identifier (TL-ID)).

No.	TL-ID	Ses-ID	# runs (# reps.)	Task outline	Main inter-action object	Varied object specifications
1	c01	01	2 (1)	Neutral pose, calibration pose, neutral pose		
2	e01	02	1 (5)	Elevate an item onto a table, lowering it down again	***imag***. **tool** **case**	w = **3 kg**
3	e02	– ” –	1 (5)	– ” –	**tool case**	– ” –
4	e03	– ” –	1 (5)	– ” –	***imag***. **tool** **case**	– ” –
5	e04	– ” –	1 (5)	– ” –	tool case	w = **2 kg**
6	e05	– ” –	1 (5)	– ” –	– ” –	w = **1 kg**
7	g01	01	1 (5)	Reach-grasp-relocate action with trunk rotation	can	d = **p-def**.
8	g02	– ” –	1 (5)	– ” –	– ” –	d = **60 cm**
9	h01	01	1 (5)	Reach-grasp-handover-relocate action with hip flexion	can	d = **p-def**.
10	h02	– ” –	1 (5)	– ” –	– ” –	d = **80 cm**
11	o01	02	1 (5)	Open and close a door	**door**	
12	o02	– ” –	1 (5)	– ” –	***imag***. **door**	
13	o03	– ” –	5 (1)	Approach, open, and pass a door (**normal** speed)	**door**	
14	o04	– ” –	5 (1)	– ” –	***imag***. **door**	
15	o05	– ” –	5 (1)	Approach, open, and pass a door (**in a hurry**)	**door**	
16	o06	– ” –	5 (1)	– ” –	***imag***. **door**	
17	r01	02	1 (5)	Hanging and retrieving an object from a hook	**towel**	h = **shoulder**
18	r02	– ” –	1 (5)	– ” –	**jacket**	– ” –
19	r03	– ” –	1 (5)	– ” –	**towel**	h = **eye**
20	r04	– ” –	1 (5)	– ” –	**jacket**	– ” –
21	r05	– ” –	1 (5)	– ” –	**towel**	h = **≥ knee**
22	r06	– ” –	1 (5)	– ” –	**jacket**	– ” –
23	r07	– ” –	1 (5)	– ” –	**towel**	h = **overhead**
24	r08	– ” –	1 (5)	– ” –	**jacket**	– ” –
25	s01	01	2 (1)	5-repetition sit-to-stand test (5RSTST)		
26	u01	01	2 (1)	Upper limb task with controlled object lift	can	
27	w01	01	1 (5)	Walk in a **normal** speed		
28	w02	– ” –	1 (5)	Walk **in a hurry**		
29	w03	– ” –	1 (5)	Walk in a normal speed	**backpack**	
30	w04	– ” –	1 (5)	– ” –	**bottle crate**	

The table includes a session identifier (Ses-ID), the total number of runs, and the number of action repetitions (reps.) recorded in each run. Blocks of related tasks share the same starting letters, i.e., different variations of similar tasks (marked in boldface). Task variations include the execution speed, type of objects ("imag.” for imaginary objects), and other object specifications. The object weight (w), distance (d; “p-def.” denotes a participant-defined distance to an object), and height (h) were varied in specified tasks.

#### Calibration and neutral poses (c01)

To ensure high-quality MoCap data, the calibration pose is imperative for individualized system configuration to enable accurate tracking of the movements of each participant. As such, this pose establishes a baseline for each participant and was assessed prior to each individual recording as part of the MoCap recording scheme. In order to document the execution of the calibration pose, participants held the neutral pose, the calibration pose, and then again the neutral pose for about 2 seconds each (see Table 2, no. 1). The calibration pose was executed as the “0-position” described by Ryf and Weymann^76^. It required participants to stand upright with their arms down the side of their bodies, palms facing the thighs, fingers straight, thumbs pointing forward, and feet pointing straightforward with a spacing of about a foot’s width (15 cm) between them. The head and gaze were directed forward. Besides the calibration pose, the neutral pose is crucial as the initial position for all movement tasks. It closely resembles the calibration pose, but encourages participants to assume a natural and comfortable standing position, resulting in more variability in wrists and finger positioning, increased flexion in the hips and knees, and a more relaxed torso posture.

#### Elevating tasks with real and imaginary toolcases of varying weights (e01–05)

These tasks focus on the movements of elevating (lifting) plastic tool cases of varying weights (1 to 3 kg) and the simulated movements of elevating an imagined (imaginary) tool case without the actual physical objects (see Table 2, no. 2–6). The simulated movements of elevating the imaginary tool case were referenced to a 3 kg dumbbell presented as a reference weight prior to executing the tasks. The objective of this category of tasks was to capture participants’ movements of elevating both physically present and imaginary tool cases. The MoCap data recorded with these tasks can provide insights into the potential effects of weight variability and the presence or absence of physical objects on the biomechanics of the movements. Notably, tasks involving imaginary tool cases were recorded both before (e01) and after (e03) the interactions with the actual physical tool cases (see Table 2).

#### Object placement at table height with trunk rotation (g01–02) or trunk flexion and self-handover (h01–02)

Tasks g01–02 (see Table 2, no. 7–8) required the participants to perform two variations of an object placement task involving reaching and grasping a cylindrical can (7.2 cm wide, 19.3 cm tall, 500g) that was placed at the level of table height. These tasks focus on axial trunk rotation, which plays a crucial role in gait^77,78^ and movements of reaching sideways. Reduced range of motion (ROM) of the pelvis during axial trunk rotation, as seen with aging^79^, can influence balance dynamics and postural control when performing reaching tasks from a standing position. To execute the task, the participants stood with their feet about a foot apart, weight evenly distributed in the same position. The can was initially placed at position A to the left side of the participant (see Supplementary Information for spatial setups of this task and recording space, Fig. S1b and Fig. S2, at recording spot 4). Participants started from a neutral pose, reached for the can with the right hand, grasped and transported it over the midline to position B, and returned to neutral pose. Afterwards, the movement sequence was also performed with the left hand, moving the can from position B back to position A. The action involving movements in both directions was executed five times. The correct grasping type was explained and demonstrated during the task instruction. Participants were instructed to maintain a moderate speed, avoid lifting their feet, keep their knees straight, briefly return to the neutral pose between phases, and look straight ahead when performing the movement. In task g01, the reaching distance to the can was individually adjusted for each participant. For g02, the reaching distance was standardized for all participants (i.e., distances 01 and 02 in Supplementary Information, Fig. S1b were set to 60 cm).

Tasks h01–02 involve self-handover motions. The participants were instructed to challenge forward flexion to engage postural control while integrating a self-handover motion during the object placement task (see Table 2, no. 9–10). The can was positioned anterior-laterally on a table at position B to the right side of the participant (see Supplementary Information, Fig. S1c) and was transferred to position A with a controlled handover as the can crossed the median line. The test protocol was similar to g01–02, with movements performed at an individualized comfortable reaching distance (h01) and at a predefined, standardized reaching distance of 80 cm to the object (h02; see Supplementary Information, Fig. S1c).

#### Interaction tasks with real and imaginary doors (o01–06)

Opening, closing, and passing through a door are typical daily tasks^80^ that are essential for unrestricted locomotion and are commonly included in datasets reflecting daily activities^46,81,82^. The door interaction tasks aimed to investigate human movement patterns in the context of interacting with the environment (see Table 2, no. 11–16), with a focus on individualized measures of anthropometry and spatial distances (see spatial measures in Supplementary Information, Fig. S1d). The open-close action with the door being either physically present or mentally imagined was first captured in isolation (at recording spot 6, see Fig. S2 in Supplementary Information). Afterwards, these actions were combined with walking sequences (starting from recording spot 7, see Fig. S2 in Supplementary Information). The first task in this category (o01–02) required reaching for the lever-style door handle, opening the door to reveal a viewing target in the room behind it, and closing the door. Subsequently, the task was extended by approaching the door within three steps and walking four further steps after passing through it, with the movements recorded at either a self-determined normal (o03–04) or fast walking pace (o05–06). The tasks with an imaginary door (see Table 2, o02, o04, and o06) closely resembled those performed with the real door (i. e., o01, o03, and o05); however, they required the participants to mentally simulating the door opening tasks and executing them without the physical context of the objects (e.g., height and shape of the door handle, space restrictions associated with the door moving along its hinge). Participants were provided with an image of the door handle and could orient themselves based on surrounding environmental cues, such as the distance to the (presumed) door. To minimize potential priming effects, the tasks with the imaginary door were only recorded once prior to performing movements with the door being physically present. Both sets of movements were recorded at the same locations and with an identical spatial setup.

#### Targeted reaching and object interactions at various heights (r01–08)

**Fig. 3:**
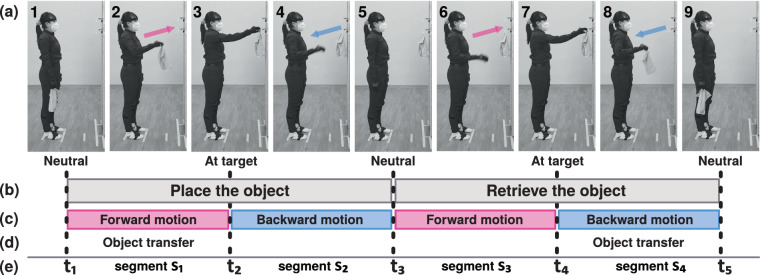
(**a**) Schematic images showing one movement repetition of the task (r01) of placing a towel on a hook and retrieving it. (**b**) The movement consists of two actions—placing and retrieving—and incorporates (**c**) motion in two directions, i.e., either forward to reach towards the target or in reversal to return to the neutral pose (see also color-coded arrows in (**a**) indicating motion direction). (**d**) Moreover, each action contains a phase where the object is transferred, either during forward motion *until* the target (hook) is reached for hanging the object, or during the reversal motion *after* the hook was reached to retrieve the object. (**e**) For data analysis, the time frames *t*_1_ − *t*_5_ are detected that divide the recorded data into motion segments (i.e., the placing action into segments *s*_1_ and *s*_2_, and the retrieving action into segments *s*_3_ and *s*_4_). Such movement segments allow for an analysis of the motion execution in relation to the motion direction and object involvement.

Tasks in this category involved placing an object on a hook and subsequently retrieving it. These reaching movements were carried out at individualized heights (ranging from approximately knee height to overhead height with the need for standing on tip-toes) and involved two different objects (a towel and a child-sized jacket), as detailed in Table 2 (see no. 17–24). The participants began by fully extending their right arm, reaching to hang the object on a hook at the individualized person-specific heights and distances (see spatial setup configuration in Fig. S1e in Supplementary Information). With a controlled and deliberate motion, participants maneuvered the arm to secure the object on the hook and returned to neutral pose (see Fig. 3a,b, “Place the object”). Following this, they re-started from the neutral pose, raised the right arm to lift the object off the hook and ended in neutral pose (see Fig. 3a,b, “Retrieve the object”).

#### 5RSTST (s01)

A 5-repetition sit-to-stand test (5RSTST), also known as timed chair stand or five times chair rise test, was also included in the present dataset, following the procedure established by Bohannon *et al*.^75^. Participants were instructed to rapidly stand up from a chair to the standing posture and repeat this movement for five times without stopping between repetitions, while keeping both arms folded across their chest (see Table 2, no. 25). While seated, participants were instructed to place their entire body weight on the chair without bending their trunk forward. When standing up, they were instructed to fully rise with straight legs. Participants practiced these movements twice, initially at a slower pace to ensure accuracy and then at maximum speed. Two runs of the 5RSTST (each with five STS movements) were recorded (see Fig. S2 in Supplementary Information, recording spot 1). Participants were seated on a chair with a height of 45 cm from the floor. The chair had no arm rests. Movement time, measured with a stopwatch (Genutek, model CG-501, stated accuracy 1/100 s), started with the command “Go” and ended when the participants’ buttocks touched the chair after the fifth repetition.

#### Upper limb task (u01)

This task was designed to capture controlled movement of the upper extremity (see Table 2, no. 26). Participants were instructed to grasp a cylindrical can (same as in tasks g01–02 and h01–02) with the right hand that was positioned adjacent to them. Subsequently, while holding the object with both hands, palms facing down and arms outstretched, they lowered the object as far as possible while maintaining a neutral standing position. This was followed by an upward arm movement to shoulder height, still maintaining the grasp with both hands, before ultimately lowering and returning the object to its initial position. The execution was initiated and ended in neutral pose.

#### Walking (w01–04)

Walking tasks serve as a common framework for assessing basic physical and neurological health of the elderly, such as the early detection of cognitive decline^83,84^ and the risk for falling^8,85–89^. Four walking tasks, each with five repetitions, were recorded (see Table 2, no. 27–30). These tasks varied in walking speed and object interactions, including: w01 – walking at a normal speed, w02 – walking in a hurry, w03 – walking at a normal speed while carrying a backpack on the back, and w04 – walking at a normal speed while carrying a bottle crate with both hands. The walking movements were recorded along a fixed path, spanning a length of 3.55 meters (see Fig. S2 in Supplementary Information, recording spots 2 and 3). The recording started with participants standing in neutral pose (at spot 2). They walked to the end of the path (spot 3) at the instructed speed, keeping their gaze straight ahead. When reaching the end of the path (spot 3, marked on the floor), they turned around until both feet were positioned in front of the marked line, then returned to the start (spot 2), and turned around to face the initial starting position in neutral pose. This sequence was repeated five times. To encourage a natural gait pattern, participants were informed that precise alignment with the marked lines was not essential; rather they were instructed to briefly stand stably with both feet at the starting and turning points before resuming walking. Participants imagined being late for an important appointment during the hurried walking task (w02). In the carrying task, the participants adjusted the straps of the backpack to their individual comfortable level (w03). Calibration poses were done without objects to ensure correct body segment orientation. Before each task, a practice run—typically one trial—was conducted, with additional repetitions allowed if needed.

### Acquisition setup and procedure

To prevent participant fatigue, each participant visited the lab on two separate recording days for two data collection sessions. Each session was limited to three hours with short breaks in between tasks as needed. Participants were instructed to wear snug sports clothing for unrestricted movement and easy access to the shoulder area and right knee for anthropometric measurements. A range of anthropometric measures were utilized to create individualised actor body profiles^90–92^ in the MoCap software Rokoko Studio to ensure precise data alignment with each participant’s body dimensions. Additionally, to account for natural variations in movements, the experimental setup for object interaction tasks was customized for each participant based on their individual anthropometric characteristics (see Movement tasks above and Supplementary Information, Section 3). All anthropometric and spatial measurements were taken while wearing personal clothing, with the MoCap data suit (soft textile with integrated IMU sensors) worn over it for the MoCap data collection.

A training phase was conducted to familiarize the participants with the postures and procedure of the MoCap recordings in a standardized manner. The participants were informed that an accurate execution of the *calibration pose* throughout the entire recording session was of utmost importance to ensure data quality.

Subsequently, the quality of the data recorded by the suit and gloves was iteratively assessed through a series of quality checks. Any issues were addressed to optimize assessment precision before the MoCap data of the movement tasks were recorded. Every task was preceded by at least one unrecorded *practice trial* which served as training to ensure correct movement execution. The experimenter provided feedback on deviations from the intended procedure, such as fidelity of the motion performance, speed and timing, and proper orientation towards marked positions. Following the recommendations by Schall *et al*.^68^, the practice trial was followed by a calibration pass in the actual recording location to ensure proper orientation of body segments. This procedure further established the actual recording spot (see Fig. S2 in Supplementary Information) as the coordinate origin of the positional MoCap data, ensuring correct relative positional coordinates for data acquisition. The MoCap data was recorded in the same fixed order for all participants: in the first recording session (Ses-ID 01 in Table 2), task categories w, s, g, h, c, and u; and in the second session (Ses-ID 02 in Table 2), task categories r, o, and e. The same task order was used for all participants to avoid potential order effects that may confound analyses of age-related or individual differences^93^. In each MoCap recording, the *neutral pose* was utilized as starting and ending pose for each trial, except in tasks where a carried object or a seated posture made the use of the neutral pose impractical (see Table 2, s01 and w03–04).

### Data pre-processing

The MoCap acquisition software provides a range of data pre-processing capabilities aimed at improving data quality. In this software, an individual’s recorded motion data is represented through a virtual avatar in 3d space. By applying filters, the global avatar’s position or the positioning of selected body segments (such as the feet) is automatically adjusted. Given that the magnetometers are sensitive to magnetic disturbance in the recording space^94,95^ and the gyroscopes to fluctuations of temperature^96,97^, there could be drifts in the recorded data because of these^68^. A “drift-correcting filter” was applied to mitigate noise in the data due to such global drifts (except for walking tasks towards and through doors, i.e., o03–06 in Table 2). This filter adjusted the global end position to align with the global start position of the data for movement tasks where the recordings began and ended in the same location, thereby correcting global drift in the recorded positional data over time. Additionally, a “locomotion filter” was applied to all data files to estimate contact of the feet with the ground^98^. Since the data suit lacks sensors to record toe movements (representing only general feet movement, see Fig. 1a), the “toe bend filter” was applied to all files to simulate the natural bending of the foot upon ground contact. For movements involving standing on tip-toes, such as reaching for targets at higher heights (r07 and r08; see Table 2), the “toe bend filter” was used to keep the feet at ground level (by applying the option “feet above floor”). This procedure corrected the vertical feet positioning to avoid penetrating the ground level by adjusting the rotation of the thigh, knee, and ankle using inverse kinematics^98^. Among the 1568 recordings (32 participants each with 49 recordings), only five were removed due to specific issues (see Section 4 in the Supplementary Information for details on removed recordings), indicating smooth and successful data recording.

## Data Records

**Fig. 4:**
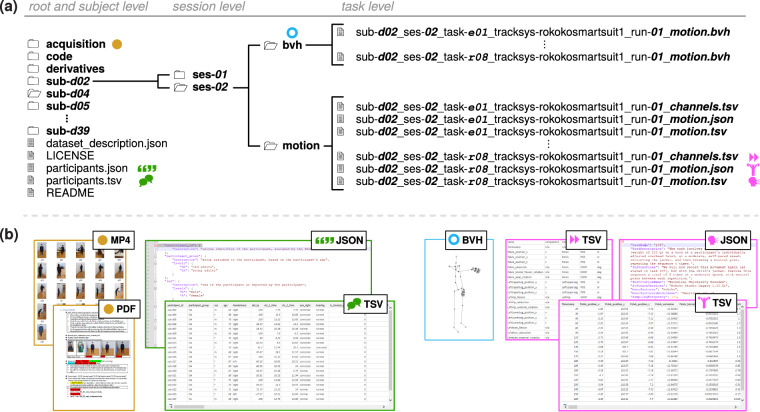
Folder structure and associated files of the *CeTI-Age-Kinematics* dataset adhering to the BIDS standard^100,101^. (**a**) The structure displays the folders and files at the root level (left-most column), the session level (2nd column), and the task level (3rd column) of the data dictionary. Colored symbols in (**b**) relate to excerpts of exemplary meta and kinematic MoCap files.

The dataset is available in a figshare repository *CeTI-Age-Kinematics*^99^. The data records are structured according to the Motion-Brain Imaging Data Structure (BIDS) standard^100,101^ (Version 1.9.0^102^), as shown in Fig. 4. In addition to the standard files (marked with green and pink symbols in Fig. 4), the dataset also includes: (i) files describing the process of data acquisition (marked with a yellow dot in Fig. 4) such as exemplary videos demonstrating task executions as well as data collection protocols, e. g., acquisition_protocol_ses-01.pdf, and (ii) BioVision Hierarchical MoCap data (BVH) files for visualizing and processing motion data in 3d animation software or BVH viewers (marked with a blue ring in Fig. 4).

### Folder structure

Motion-BIDS standardizes the folder structure, data formats and metadata structure using JavaScript Object Notation (JSON). Starting from the root directory ceti-age-kinematics/, it utilizes the following file organisation (see Fig. 4a; other detailed technical documentation are available in previous reports^100,102^):

- acquisition/ subfolder stores the acquisition protocols and example videos relevant to the entire dataset.
- code/ subfolder contains all essential source code (see also Code Availability for details), including environment setup files, Python scripts for reproducing validation approaches, and helper scripts for shared routines like loading data and managing figures.
- derivatives/ subfolder provides processed data, including the processed files after applying a segmentation processing pipeline (see Technical Validation), analysis results for creating the graphs, and stored images generated from the source code.
- participant.tsv and participant.json files describe the participants’ characteristics (see green symbols in Fig. 4a) such as demographic, anthropomorphic, and other data (as detailed later in this section).
- dataset_description.json and README.md files provide metadata and additional information on the dataset.

All other data is organized in the participant-specific subdirectories.

- Participant subdirectories (sub-d02/, etc.): Each participant has their own subdirectory that is named with a unique identifier, e. g., sub-d05/ for the participant 5.
- Session subdirectories (ses-01/ or ses-02/): Within each participant’s folder, there are subdirectories for each recording session, such as ses-01/ for session 1 (referred to as Ses-ID in Table 2).
- MoCap data subdirectories (motion/ or bvh/): In each session subdirectory, the MoCap data is stored in Tab-separated values (TSV) format in the motion/ subfolder and in the BVH format in the bvh/ subfolder (see further details in next section).

### Kinematic MoCap Data

In this dataset, the participant’s kinematic movement data is saved in two different formats: TSV and BVH, each serving distinct purposes. The TSV format stores sensor data in a structured tabular form, allowing for detailed sensor data analysis. It is accompanied by relevant metadata files, ensuring adherence to the Motion-BIDS standard^100–102^. The BVH format stores MoCap data in a hierarchical structure and is commonly used for animation and visualization purposes^103,104^. The TSV files include the positional and rotational sensor data from both the data suits and gloves (see Table S3 in Supplementary Information for an overview of sensor labels). Specifically, they contain information from 17 of 19 sensors of the data suit, excluding those of the hands (see Sensor identifiers (S-IDs) 13 and 18 in Table S3 in Supplementary Information). Wrist motion in the TSV files is recorded from the data gloves (see S-IDs 20 and 26 in Table S3 in Supplementary Information). Finger data (S-IDs 21–25 and 27–31 in Table S3 in Supplementary Information) is included only in the BVH data (see Table S4 in Supplementary Information), which represents motion hierarchically, preserving finger articulation. TSV files store joint data in a flat tabular format to simplify storage and processing. They are well-suited for ML pipelines by allowing for high-level motion analysis without encoding detailed skeletal dependencies such as finger movements.

These two forms of MoCap data are stored respectively in the motion/ and bvh/ subfolders and follow a file naming template according the Motion-BIDS standard^102^: sub-<subject_label>_ses-<session_label>_task-<task_label>_tracksys-rokokosmartsuit1_run-<run_index>_motion.{tsv∣bvh}. Table 2 states the <session_label> Ses-ID, the <task_label> TL-ID and the number of runs recorded (see “# runs”). While most of the tasks include only one run, seven tasks (see c01, o03–06, s01, u01 in Table 2) contain multiple runs that can be accessed separately by specifying the <run_index> denoted by a number between 01 and 05, depending on the task. This results in a total of 49 recordings of MoCap data (in either TSV or BVH format) for each participant (see Section 4 in the Supplementary Information for the number of runs by task).

#### TSV data

**Fig. 5:**
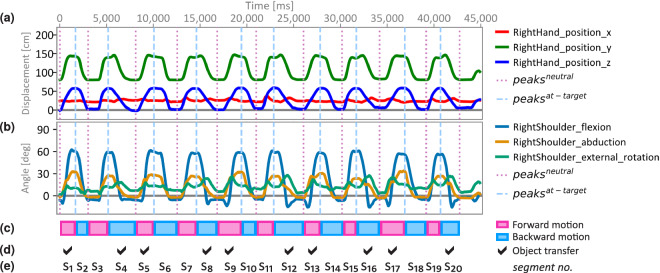
Raw MoCap data over time in milliseconds [ms] of one participant (Id 31, YA, male) while performing task r01 (see no. 17 in Table 2), displaying either (**a**) displacement of (*x*, *y*, *z*) positions of the RightHand in centimeters [cm], or (**b**) anatomical orientation of the RightShoulder sensor in Euler degrees [deg]. This reaching task is compound from (**c**) forward and backward movements (cf. Fig. 3) and (**d**) involves phases with and without object transfer. The vertical lines in (**a**)–(**b**) mark the points in time that were detected via peak detection (see the Validation section and Supplementary Information, section 8 for details) and (**e**) can be utilized for subdividing the raw MoCap data into multiple motion segments.

Each participant’s movement data is structured into three distinct files per task (see pink symbols in Fig. 4): The _motion.tsv file contains the raw sensor data capturing the participant’s movements. The _channels.tsv file provides a description of the sensor data, specifying the column names, types, and measurement units of each sensor. Additionally, the _motion.json file includes metadata, such as a task outline and instructions provided to the participants. Figure 5 shows the positional and rotational data of one exemplary joint in a raw TSV MoCap file.

- Positional data (see Fig. 5a) describe the position of the center of mass of the relevant body segments in centimeters, denoted as position_{x∣y∣z} in absolute coordinates (see segment labels in Table S3 in Supplementary Information). The coordinate system for positional data is established during calibration, with the origin of the coordinate (0, 0, 0) set to the standing position of the participant, and the direction of coordinate axes aligned with the anatomical planes. The *x*-axis aligns with the frontal (coronal) plane for representing the medial-lateral motion (left-right), the *y*-axis aligns with the sagittal plane for representing superior-inferior (up-down) movements, and the *z*-axis aligns with the transverse (horizontal) plane for representing anterior-posterior (forward-backward) movements.
- Rotational MoCap data is measured as joint angles in degrees (see Fig. 5b) for the joint labels listed in Table S3 in Supplementary Information. The centers of these joints and the directions of movements (using the corresponding medical terminology, e.g., terms such as extension, axial_rotation, etc.; see Table S3 in Supplementary Information) are defined according to the International Society of Biomechanics (ISB) standard^105–107^. The calculation of rotational data relies on Joint Coordinate Systems, which are tailored to each of the joints to represent joint angles as relationships between the axes of body segments connected by a joint (see Section 4 in the Supplementary Information for further explanations).

#### BVH data

BVH files contain MoCap data for processing in a 3d modelling and animation software^104^ (see blue donut symbol in Fig. 4). They denote the movements of the sensors according to a hierarchical skeleton structure^103^. Specifically, the current dataset follows the Maya inverse kinematic (IK) skeleton structure^108^. We chose this skeleton structure because it ensures best interoperability, e.g., with MotionBuilder^109^ or Blender for character animation, or OpenSim for biomechanical simulation^110^. The mapping of the IMU sensor data to the Maya IK skeleton structure in a BVH file is shown in Table S4 in the Supplementary Information.

### Demographic, anthropometric, and other data

In accordance with the specifications of the BIDS standard^101,102^, the file participants.tsv includes all data related to the characteristics of the participants, with participants.json providing meta information on the TSV column names, their values, measurement units, and a column description (see green symbols in Fig. 4). The participants.tsv file contains information for each participant, with a total of 201 columns. These columns contain variables grouped into six categories: basic and extended demographic data, anthropometric body measures, individualized spatial configurations, motion tracking quality, and further demographic data (see Table S5 and associated descriptions in Section 6 of Supplementary Information for details).

## Technical Validation

To assure the integrity and reliability of the data, we conducted technical validations in several steps. Results from selected key analyses are presented below, after an overview of the approaches we took.

### Overview of validation analyses

**Fig. 6:**
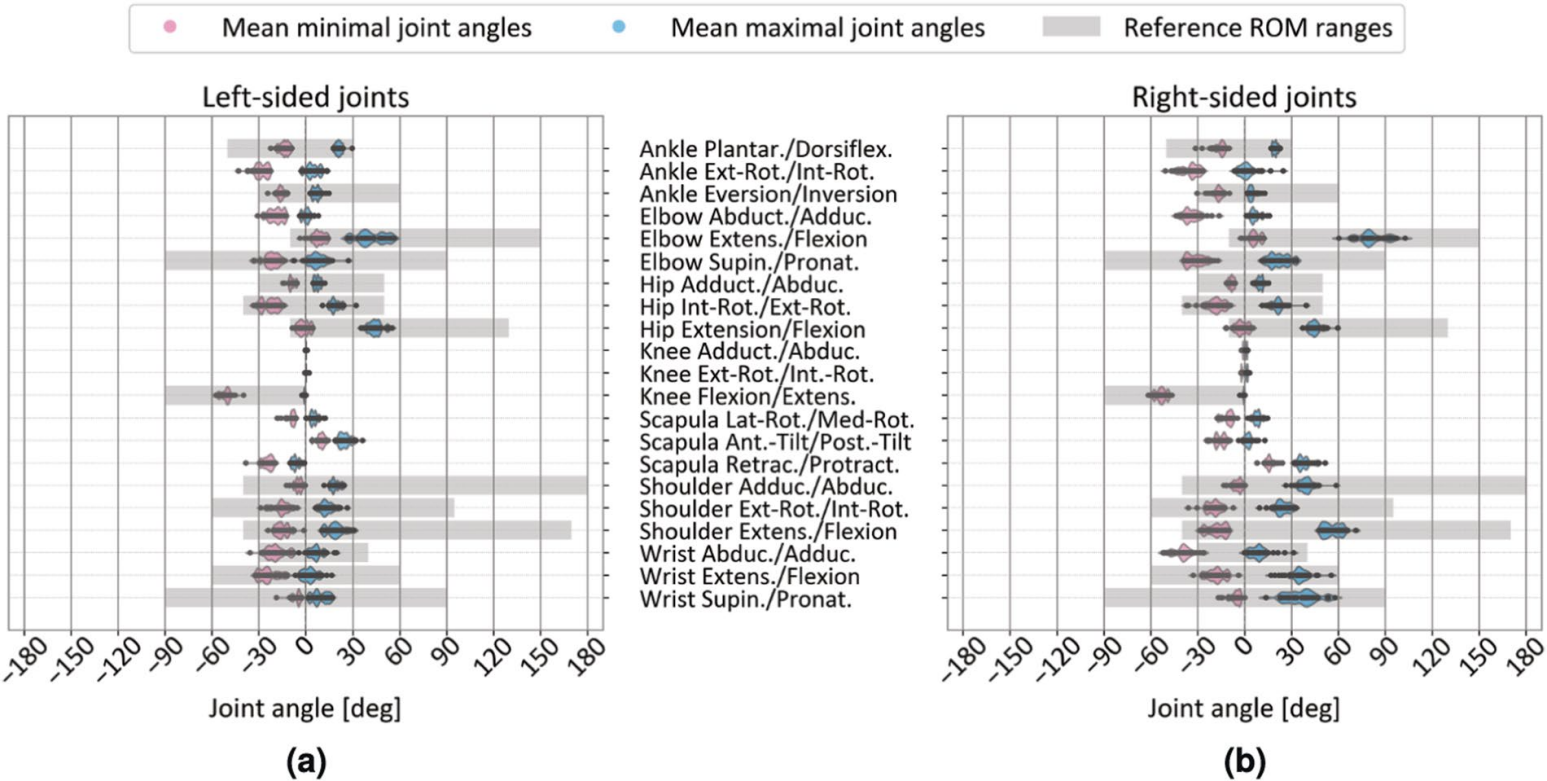
Joint angle distributions over all participants, showing the mean peak minimal (in pink) and mean peak maximal (in blue) joint angles, separated for joints on (**a**) the left side and (**b**) the right side of the body. Anatomically typical range of motion (ROM) reference ranges (shaded in gray) are taken from Ryf and Weymann^76^.

As a first step we verified that the recorded data correspond both anatomically and biomechanically to human motion characteristics of daily movements. This was assessed by showing anatomically typical joint angle distributions across all participants and recordings (see Fig. 6), as well as during the standardized calibration pose (c01 in Table 2; results of validating recordings of the calibration pose can be found in in Section 7 and Fig. S3 in Supplementary Information). As the next step, a task-specific validation approach was applied, which tailored the method to the specific movement type while allowing for examinations of task variations, such as target height and object type to validate the recorded data. Different types of movements exhibit distinct spatial, temporal, and dynamic properties that require different analytical approaches. For example, in walking movements the analysis can focus on parameters of the human gait cycle such as stride time and stride length^83,111^; while in reaching tasks the analysis may emphasize velocity profiles and shape characteristics of 3d reaching trajectories^112–114^.

**Fig. 7:**
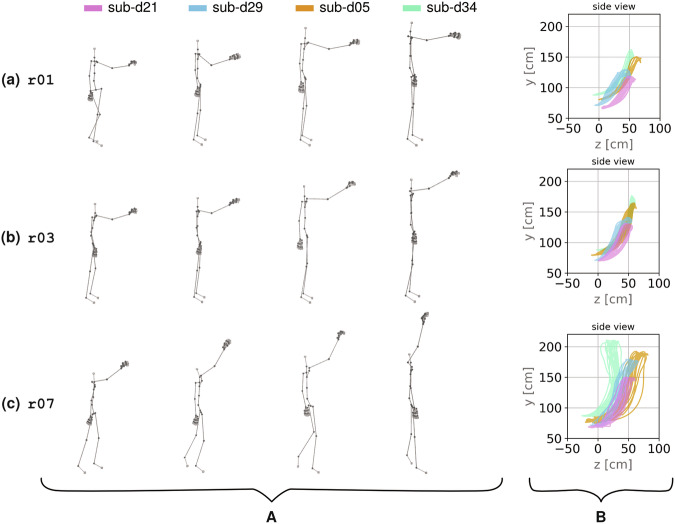
BVH skeleton representation of four selected participants (panel A) and their corresponding spatial reaching trajectories (panel B, color-coded by participant ID) across three reaching tasks. The skeleton representation (panel A) shows a moment when participants reached for the hook to place a towel, either at (**a**) shoulder height, (**b**) eye height, or (**c**) a self-defined overhead height. The plots display participants sorted by their overall body height (stature) and participant group (YA or OA), beginning with the shortest participants (sub-d21, OA, and sub-29, YA, both female) and ending with the tallest participants (sub-d05, OA, and sub-d34, YA, both male). The spatial reaching trajectories (panel B) display the raw MoCap data for each participant (color-coded by participant ID) and tasks (a-c), with varying reaching heights reflected on the y-axis.

**Fig. 8:**
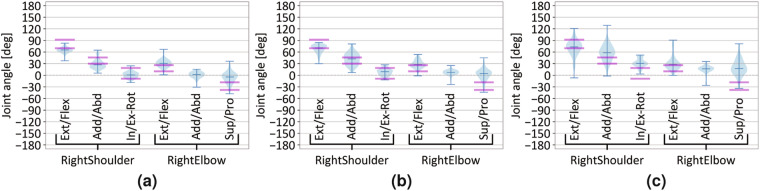
Joint angle distributions of the right shoulder and elbow sensor (see S-IDs 16–17 in Table S3 in Supplementary Information) across all participants in reaching tasks (see Table 2). For (**a**) r01 (shoulder level), (**b**) r03 (eye level), and (**c**) r07 (overhead); the distribution of joint angles of the right arm is shown for moments in time when participants reached the target (denoted with “at target” in Figs. 3 and 5). Reference values, depicted in pink, were taken from Vandenberghe *et al*.^123^.

For task-specific validation, we first conducted a visual analysis of the effect of height variations in specific (unprocessed) reaching tasks (i.e., r01, r03 and r07 in Fig. 7), utilizing the TSV and BVH of four selected participants. Next, we employed a processing segmentation pipeline to effectively segment these distinct reaching tasks (i.e., r01-03, and r07 in Table 2, see Supplementary Information, Section 8, cf. Fig. S4 in Supplementary Information). Based on the thus pre-processed data, we provide assessments of the quality of the data in joint angle distributions for reaching tasks involving varied target heights (see Fig. 8, at r01: shoulder, r03: eye, and r07: overhead level).

**Fig. 9:**
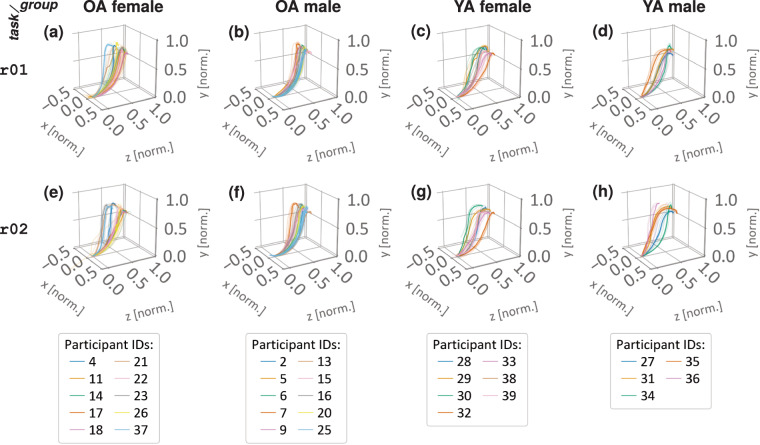
Reaching trajectories in 3d space for task r01 (top row) and r02 (middle row), categorized by age (older adults (OA): (**a**) & (**b**) versus younger adults (YA): (**c**) & (**d**)) and sex (female: (**a**) & (**c**) versus male: (**b**) & (**d**)). Each bold motion trajectory represents the mean (*x*, *y*, *z*) position values of the right wrist for the motion segments including the forward-upward reach (see segment 1 in Fig. 3e) with the respective object (r01: small towel, r02: child’s jacket). Faint lines depict the five individual repetitions for each each individual participant. The color-coding corresponds to the participant ID.

Taking also a third approach of validation, we examined the spatial 3d trajectories of selected reaching movements interacting with different objects (involving for tasks r01: a small towel, and r02: a children’s jacket, see Table 2) across participants, considering different age groups and sexes (see Fig. 9).

### Anatomically typical joint angle distributions over all tasks

We examined the sensor data to ensure that the joint angles fell within the typical ROM achievable by humans (see Fig. 6). Specifically, we assessed the peak minimal and peak maximal joint angles for each recording, resulting in two matrices with each matrix containing 1,563 × 51 values that denote the angles for 17 joints in each 3 anatomical directions (see Table S3 in Supplementary Information) for each single recording (see Data Records). We then calculated the *mean* minimal and maximal peak values by averaging over 49 recordings (or fewer if missing, see Data pre-processing) for each of the 32 participants. The mean minimal and maximal values were stored in two 32 × 51 matrices, stating one mean minimal (or maximal) joint angle for each of the 17 joints and their respective motion directions for all 32 participants. Next, we displayed the distribution of mean minimal (in pink) and mean maximal (in blue) joint angles across participants for joints located on the left (see Fig. 6a) and right (see Fig. 6b) body side, including the ankle, elbow, hip, knee, scapula, shoulder, and wrist joints. This approach highlights the different utilization of the left and right body joints, especially of the upper limbs, among participants in our samples. Median-centred values for joints such as the head, thorax, and pelvis are available in the derivatives folder (see Data Records) and can be reproduced by using our documented source code (see Code Availability, script starting with 03). The movement directions and ranges of joint angles are defined according to the ISB standard^105–107^ in Fig. 6. Furthermore, we included reference ranges of anatomically feasible ROM as defined by Ryf and Weymann^76^ (see gray-colored horizontal bars in Fig. 6) that are aligned with the recommendations of the ISB, using the zero neutral pose as the starting point^106,107^. Notably, we added the terms describing the opposite movement direction in Fig. 6, because the original sensor labels state only one movement direction (as explained in Section 4 in Supplementary Information, see TSV joint labels in Table S3).

Generally, the sensor data in our dataset align with the expected human anatomical ranges of motion^76^. Notably, higher mean minimal and maximal values were observed for right-sided joints (see Fig. 6b), particularly in the upper limbs, such as the right shoulder, elbow, and wrist. This is not surprising, as the unilaterally executed actions (involving e01–05, o01–06 and r01–08, see Table 2) were performed with the right hand, necessitating a greater and more articulated range of movement. Previous research has shown that the ROM for upper-limb tasks during activities of daily living can require peak values for wrist abduction (radial deviation) close to the maximum anatomical limits^115^. Our analysis revealed some irregularities in wrist abduction (radial deviation) of the right hand (see Fig. 6b), with values exceeding the expected range of  − 30 degrees. These deviations were consistent across multiple participants, indicating a systematic measurement discrepancy. One contributing factor may be the inconsistent execution of the calibration pose, particularly regarding wrist posture, as illustrated in the violin plots in Fig. S3 in Supplementary Information: there is notable variability in the wrist orientation during calibration, particularly in the abduction/adduction parameter for both left and right wrists, as well as in the flexion/extension of the left wrist alone (as discussed in Supplementary Information, section 7). This suggests a relationship between calibration variability and the observed deviations in wrist abduction/adduction in Fig. 6b. In terms of the other anatomical directions of the wrist and the ROM of other joints, no notable peculiarities are apparent in Fig. 6, suggesting a good overall motion reproduction of the MoCap technology within this dataset.

### Visual analysis of skeletal representation and reaching trajectories across height variations

Given that this dataset encompasses various task variations with regard to object position and type, we conducted a visual validation of the effects of height variations in selected reaching tasks. The plots in Fig. 7 (panel A) show the BVH skeleton representations of four selected participants at a moment in time when they reached for a hook to place a towel (cf. Fig. 3a, image no. 3), either at shoulder height (see Fig. 7a, cf. r01 in Table 2), eye height (see Fig. 7b, cf. r03 in Table 2), or a self-defined overhead height (see Fig. 7c, cf. r07 in Table 2). The selected participants represent the shortest and tallest individuals of each participant group (see participant characteristics in the Supplementary Table S1). They are displayed in order of height, starting with the shortest participants (sub-d21 in pink, OA, and sub-29 in blue, YA, both female), followed by the tallest participants (sub-d05 in orange, OA, and sub-d34 in green, YA, both male).

Panel A of Fig. 7 reflects the differences in body height across participants, with the skeleton representation showing increasing height from left to right in three rows (a, b, c) for three reaching tasks at different heights. The variations in reaching heights are evident from the vertical reach (i.e., the positioning of the hands at different elevation levels in 3d space), which is located at each participant’s individual shoulder height (see Fig. 7a), head height (see Fig. 7b), or overhead height (see Fig. 7c). The visualizations in panel A of Fig. 7 further shows individual postural differences, such as variations in right elbow flexion, different hand postures of the active (right) and inactive (left) hands, varying orientations of the shoulder girdles towards targets, and diverse hand positioning relative to the hips and spine. Furthermore, Fig. 7c reveals differences in the length of forward steps towards the target and the choice of the standing foot (sub-d21 utilized the left foot, whereas all other participants the right foot, all four participants were right-handed, cf. Supplementary Information, Table S1). These individual differences in postures align well with the individual spatial configurations of the reaching tasks (see Supplementary Information, Fig. S1e). Spatial adjustments led to different orientations and (reaching) distances towards the target, affecting overall posture.

The panel B of Fig. 7 shows the motion trajectories of the RightHand_position of all four participants, color-coded by the participant ID. The visualization of reaching trajectories focuses on the right wrist sensor data (see S-ID 26 in Table S3 in Supplementary Information), as hand and wrist motion significantly contributes to the execution of daily tasks^115^. The analysis of 3d hand or wrist trajectories is a typical approach in neurocognitive research^116,117^, ergonomics^118^, and post-stroke UL rehabilitation^119^. Additionally, motion trajectories are vital for trajectory planning in robotics^120^. Determining the position and orientation of the end-effector (presented by the hand in a human execution) relative to the target is essential for developing robust motion planning algorithms. The positional data in panel B of Fig. 7 reflects the position of the center of mass of the hand segment (see Data Records), showing only the (*z*, *y*) positions from the TSV files for clarity. The coordinate origin and axes for capturing IMU position data are established during calibration, with the *y*-values typically reflecting upward-downward movements and *z*-values for forward-backward movements (see Data Records). Specifically, trajectories in panel B of Fig. 7 display the movement paths of the right wrist of four participants during task execution, as it moves up or down along the vertical *y* axis as well as towards or away from the reaching target along the *z* axis. During motion execution, the wrist alternated between two key poses: (i) neutral pose with the right hand hanging relaxed besides the body (cf. Fig. 3a, image no. 1), characterized by a *z*-position near zero, and a *y*-position corresponding to the wrist’s height relative to the floor, and (ii) a pose with the right arm extended forward and pointing towards the target (cf. Fig. 3a, image no. 3), reflected in larger *z* and *y* values of the wrist in comparison to neutral pose. Since the reaching movement is repeated five times (see Fig. 5), multiple trajectories for each participants are displayed in Fig. 7a–c in panel B.

These motion trajectories across the tasks show consistent variations in vertical displacements along the *y*-axis that correspond to the three levels of reaching height, with larger displacements indicating higher reaching heights. The largest amplitude is observed in the overhead reach (see Fig. 7c, panel B). Notably, the minimal (near zero) and maximal *z*-values vary across different reaching heights, even though the actual distance to the target remained unchanged during data acquisition. Participants positioned themselves only once at the beginning of the MoCap block in front of the target (see spatial configuration in Supplementary Information, Fig. S1e) and maintained this position while completing all reaching tasks (including r01–08 in Table 2). Variations in *z*-positions may arise due to natural pose differences in motion execution, both across repetitions and during calibration. Additionally, the reaching trajectories in panel B of Fig. 7 display a systematic pattern across participants, with shorter individuals (e.g., sub-d21 in pink) showing smaller amplitudes and taller individuals (e.g., sub-d34 in green) showing larger amplitudes across all tasks (see Fig. 7a–c). Such systematic variations correspond closely to the participants’ body heights and anthropometric measures. It demonstrates that the positional data accurately reflects the expected changes in reaching targets along the *y*-axis while preserving participant-specific characteristics in the data.

### Segmentation

This dataset captures natural, compound movements that reflect real-world tasks. In this context, segmentation is a common processing step when the data contains multiple distinct motion phases or repetitions^9,18,121,122^. Such a segmentation process provides insights into the data quality, as it can reveal distinct and discernible structures within the data. For example, while the reaching trajectories presented in Fig. 7 illustrate the full recordings of the reaching tasks, it is often useful to focus on distinct phases of a given movement. As shown in Fig. 3, the structure of reaching tasks can be complex. The tasks included a placing and a retrieving action (see Fig. 3a), which can be further subdivided into forward and backward motion phases (see Fig. 3b). Additionally, during each phase, an object may be carried (i.e., transferred from one position to another) or not (see Fig. 3d, no object transfer in segments 2–3). To validate distinct motion phases of reaching tasks, we applied a segmentation processing pipeline as detailed in the Supplementary Information, section 8, to selected specific reaching tasks (see r01, r02, r03, r07 in Table 2). To facilitate further types of analyses and the development of processing pipelines other researchers may want to utilize, the source code is published alongside with the dataset (see Code Availability, scripts starting with 05a and 05b).

The results of segmentation show the anticipated number of segments and display consistent characteristics of the signal across the segments (see Figs. 5 and 9, cf. Fig. 3e). The temporal information of the detected peaks and motion segments can be further utilized in other types of data analysis. We used the temporal information of detected peaks in Fig. 5a,b to mark these time stamps in a (unprocessed) MoCap recording at which peak values were detected in the positional data. Moreover, we analyzed the joint angle distribution only for specific time stamps in the data (also derived from peak detection, cf. Fig. 5a,b), that reflected the poses when participants have reached the target (see Fig. 8). As shown in Fig. 8, the data demonstrated the to-be-expected task-specific joint angle distributions after segmentation. These findings indicate reliable recordings of the sensor data and well confirm with the execution of tasks performed by the participants. All further processing steps and validation analyses shown in the following were conducted on segmented data.

#### Task-dependent joint angle distributions

We assessed the capabilities of the sensors to accurately capture deliberate changes in the experimental setup with the rotational data. Fig. 8 displays the joint angles of the sensors from the right upper limb (see S-IDs 16–17 in Table S3 in Supplementary Information) as participants executed a forward-upward reach to either shoulder level (see Fig. 8a), eye level (see Fig. 8b), or a self-selected overhead reaching height (see Fig. 8c). The angle distributions of the RightShoulder and, to some extent, RightElbow reflect the increased reaching height in 8a and b. The angle distribution shows a comparable pattern as observed by Vandenberghe *et al*.^123^ who found that the kinematics of the shoulder and elbow joints are affected depending on the width and heights of the targets locations to be reached. Reference joint angles for reaching a target at shoulder height in neutral position (“reference reaching task (NM)”^123^) are depicted in pink in Fig. 8. On the one hand, Fig. 8a,b show that shoulder flexion increased with increasing reaching height^123^. This becomes even more apparent, when compared to the angle distribution during an overhead-reach (see Fig. 8c) that was not part of the movement tasks investigated by Vandenberghe *et al*.^123^. On the other hand, Vandenberghe *et al*.^123^ found an increase in elbow supination with raising reaching height. We assessed, on the contrary, an increase in elbow pronation with raised reaching height (see Fig. 8a–c). While the participants in the study by Vandenberghe *et al*. carried “a handle with a pointer”^123^, the majority of participants in the reaching tasks assessed in our dataset used a movement strategy utilizing elbow pronation (palm facing downwards) for lifting the object over the hook and placing it on it. Notably, the angle distribution in Fig. 8a and b shows a comparable value range as stated by Vandenberghe *et al*.^123^ for the mean values of the shoulder joint (though not elbow joint, due to the differing object involvement), despite differences in the task design. For example, we captured the participants while standing (not sitting), at a lower “above shoulder” reaching height at eye level, and with individually adjusted medial-lateral positioning in front of the target (see Supplementary Information, Fig. S1e).

Additionally, the angular data of the RightShoulder sensor in task r01 is depicted in Fig. 5b for a young male participant, showcasing the reproduction of shoulder abduction and flexion as observed in a previous study with a young male population performing a forward-upward reach [^124^, Fig. 3]. This observation suggests that the data suit effectively captures shoulder movements with high fidelity and visually aligns well with the MoCap data recorded with a marker-based system.

#### Task- and group-dependent motion trajectories

The experimental setup of selected tasks was adapted to the anatomically feasible and individually preferred range of movement for the participants. As a result, variations in standing positions and distances relative to the object or target of interest were documented (see Supplementary Information, Fig. S1e). While environmental conditions can influence movement execution (e.g. the configuration of joint angles across different tasks), certain movements such as gait^38^, target-reaching^120,125,126^, and catching^127^ exhibit consistent movement-specific spatial characteristics across repetitions and participants. To visualize this, Fig. 9 displays the mean reaching trajectories of the right wrist (see S-ID 26 in Table S3 in Supplementary Information) in task r01 (top row, 9a–d) and task r02 (middle row, 9e–h) for forward-upward reaches involving an object (see Table 2). To illustrate age- and sex-associated differences in task execution, the reaching trajectories are displayed according to the participants characteristics, organized in rows by tasks (r01 or r02) and in columns by participant group (either OA or YA) and sex (either female or male). The trajectories are color-coded by the participant ID (see bottom row in 9), with the mean trajectory across motion segments shown in bold, and individual (participant-wise) trajectories as faint, color-coded lines. Notably, the mean trajectories (bold color-coded lines) exhibit an arc-shaped pattern, reflecting the inherent nature of the movement task and thus demonstrating the participants’ ability to execute the intended movement with trajectories that align with the expected pattern. Specifically, for task r01 (9a–d) the movement starts from the lowest position when the hand is relaxed (neutral pose), progresses in a curved upward-reaching path as the towel is lifted towards the hook, slightly raises as hanging loop of the towel is guided over the hook, and finally drops once the loop is over the hook and the towel is hanging on the hook at the end point of the trajectory. However, there are individual differences. On the one hand, the variability of the position coordinates at the end of the trajectories (*x* − *y* plane) suggests differences in the initial starting location (medial or lateral) in front of the target (note that as the data is normalized this variability is only evident at the end of the trajectories in Fig. 9). On the other hand, variations in the *y* − *z* plane reflect different movement strategies employed to guide the object towards the target. Despite these differences, a distinct pattern can be observed in the underlying topology of all the trajectories. The reaching task r02 (9e–h) is essentially identical to r01, with the exception of utilizing a different object (child’s jacket). Consequently, the resulting trajectories bear a close resemblance, distinguished by minor variations. These are task-specific adaptions in the execution of the motion, e.g. changes in curvature during the final phase of the segment before approaching the hook (top row and cf. middle row in Fig. 9).

## Usage Notes

### TSV MoCap data and JSON metafiles

TSV and JSON files are widely supported across programming languages and platforms. The source code for loading and processing the kinematic MoCap data stored in TSV format is provided as part of the dataset^99^ (details listed in the section Code Availability). For compliance with the Motion-BIDS standard^100,101^, specialized libraries such as PyBIDS^128,129^ are available for processing the data.

### BVH MoCap data

For a fast, no-code visualization of the MoCap sequences, BVH files can be opened in a BVH viewer software^130,131^, also in a web application^132^. Due to its vast spread, the BVH format is commonly supported by the majority of software programs for computer animation and 3d computer graphics^104^ to create character animations. After binding the animation to a digital character, it can be exported to game engines, for example to build interactive virtual reality applications. Recently, BVH data recorded with data suits by this manufacturer was used as an input for animating human-figured children’s drawings^133^ and virtual avatars in cultural heritage applications using the Unity game engine^134^. BVH data recorded with other IMU data suits was also used for driving biomechanical simulations in OpenSim^110^.

**Table 3:** Summary of Python scripts available in the code/scripts directory.

Name of Python script	Script description and reference
01_analyze_basic_demographics	Summarizes basic demographic data of the dataset (Table 1)
02_create_plot_joint_angles_of_calibration_pose	Displays joint angle distribution during calibration (see Supplementary Information, Fig. S3)
03_create_plot_joint_angles_across_all_tasks	Shows mean minimal and maximal joint angles over all tasks (Fig. 6)
04_create_plots_reaching_trajectories_taskwise	Visualizes trajectories for specific reaching tasks r01, r03, and r07 (Fig. 7, panel B)
05a_apply_processing_pipeline	Applies a multi-step processing pipeline for segmenting specific reaching tasks r01–03 and r07 (see next script 05b)
05b_create_plots_processing_pipeline	Visualizes processing steps of segmentation pipeline on the example of reaching trajectories (see Supplementary Information, Fig. S4)
06_create_plots_raw_mocap_data_with_peaks	Shows unprocessed positional and rotational MoCap data and marks time stamps where peaks were detected during segmentation (Fig. 5)
07_create_plots_joint_angles_reaching_heights	Displays joint angle distributions of the right shoulder and elbow at moments when participants reached the hook (detected during segmentation) for specific reaching tasks r01, r03, and r07 (Fig. 8)
08_create_plots_reaching_trajectories_groupwise	Visualizes processed reaching trajectories, categorized by age and sex, for specific reaching tasks r01–02 (Fig. 9)

Scripts 01–04 use raw data while scripts 05b–08 require applying the segmentation processing pipeline first (see script starting with 05a).

## Supplementary information

Supplementary: A Full-Body IMU-Based Motion Dataset of Daily Tasks by Older and Younger Adults

## Data Availability

The source code is published as part of the Data Descriptor^99^ and replicates the results from the Validation section. It is available in the code/ folder of the dataset (see Fig. 4a). This folder contains the following files: (i) txt and yaml files to prepare the development environment, (ii) Python scripts, located in code/scripts, to reproduce the validation approaches, and (iii) helper scripts, found in code/helpers, offering shared functionalities across scripts, e.g., routines for loading MoCap files, setting parameters of the processing pipeline, and handling figures. For detailed explanations, refer to the README file of the dataset (see Fig. 4a) and comments within the source code. Table 3 provides a summary of the Python scripts (stored in code/scripts), with their corresponding functionalities and references to the material presented in this Data Descriptor. The source code was tested on Windows 11 Home using miniconda as a package manager. The implementation relies on commonly used Python libraries in ML and data analysis, e. g., numpy, pandas, scipy, seaborn, and matplotlib. To reproduce the development environment, two files are provided to specify the necessary python libraries: environment.yaml for users of the Conda package manager^135,136^ (using conda) and requirements.txt for users of the Python Package Index PyPI^135^ (using pip). The source code is documented and contains TODO comments where parameters can be adjusted. Additional source code is provided by Hanisch *et al*.^33^, who use the same recording protocol as this dataset to capture walking tasks and 5RSTST (s01 and w01–04 in Table 2). This sourcecode can complement the code provided in this data descriptor and includes the following functionalities: (i) rendering skeletal joints positions for visual inspection, (ii) segmenting the tasks into single steps (for tasks w01–04) or sit-to-stand and stand-to-sit transitions (for task s01), and (iii) preparing the data for and conducting a classification analysis of movement tasks, participant sex, and identities utilizing a Support Vector Machine.

## References

[1] Tornero-Quiñones, I., Sáez-Padilla, J., Espina Díaz, A., Abad Robles, M. T. & Sierra Robles, Á. Functional Ability, Frailty and Risk of Falls in the Elderly: Relations with Autonomy in Daily Living. *IJERPH* **17**, 1006, 10.3390/ijerph17031006 (2020). 32033397 10.3390/ijerph17031006 PMC7037456

[2] Katz, S., Downs, T. D., Cash, H. R. & Grotz, R. C. Progress in Development of the Index of ADL. *The Gerontologist* **10**, 20–30, 10.1093/geront/10.1_Part_1.20 (1970). 5420677 10.1093/geront/10.1_part_1.20

[3] Hillman, C. H., Erickson, K. I. & Kramer, A. F. Be smart, exercise your heart: Exercise effects on brain and cognition. *Nat Rev Neurosci* **9**, 58–65, 10.1038/nrn2298 (2008). 18094706 10.1038/nrn2298

[4] Vaynman, S. & Gomez-Pinilla, F. Revenge of the “Sit”: How lifestyle impacts neuronal and cognitive health through molecular systems that interface energy metabolism with neuronal plasticity. *J of Neuroscience Research* **84**, 699–715, 10.1002/jnr.20979 (2006). 10.1002/jnr.20979 16862541

[5] Prakash, R. S., Voss, M. W., Erickson, K. I. & Kramer, A. F. Physical activity and cognitive vitality. *Annual Review of Psychology* **66**, 769–797, 10.1146/annurev-psych-010814-015249 (2015). 25251492 10.1146/annurev-psych-010814-015249

[6] Cimolin, V. *et al*. Computation of Gait Parameters in Post Stroke and Parkinson’s Disease: A Comparative Study Using RGB-D Sensors and Optoelectronic Systems. *Sensors* **22**, 824, 10.3390/s22030824 (2022). 35161570 10.3390/s22030824 PMC8839392

[7] Ghoraani, B. *et al*. Detection of mild cognitive impairment and Alzheimer’s disease using dual-task gait assessments and machine learning. *Biomedical Signal Processing and Control* **64**, 102249, 10.1016/j.bspc.2020.102249 (2021). 33123214 10.1016/j.bspc.2020.102249 PMC7591132

[8] Modarresi, S., Divine, A., Grahn, J. A., Overend, T. J. & Hunter, S. W. Gait parameters and characteristics associated with increased risk of falls in people with dementia: A systematic review. *Int. Psychogeriatr.* **31**, 1287–1303, 10.1017/S1041610218001783 (2019). 30520404 10.1017/S1041610218001783

[9] Pogrzeba, L., Neumann, T., Wacker, M. & Jung, B. Analysis and Quantification of Repetitive Motion in Long-Term Rehabilitation. *IEEE J. Biomed. Health Inform.* **23**, 1075–1085, 10.1109/JBHI.2018.2848103 (2019). 29994665 10.1109/JBHI.2018.2848103

[10] Russell, J., Inches, J., Carroll, C. B. & Bergmann, J. H. M. A modular, deep learning-based holistic intent sensing system tested with Parkinson’s disease patients and controls. *Front. Neurol.* **14**, 1260445, 10.3389/fneur.2023.1260445 (2023). 38020624 10.3389/fneur.2023.1260445 PMC10646321

[11] Raz, N. *et al*. Regional brain changes in aging healthy adults: general trends, individual differences and modifiers. *Cerebral cortex* **15**, 1676–1689, 10.1093/cercor/bhi044 (2005). 15703252 10.1093/cercor/bhi044

[12] Troke, M., Moore, A. P., Maillardet, F. J. & Cheek, E. A normative database of lumbar spine ranges of motion. *Manual Therapy* **10**, 198–206, 10.1016/j.math.2004.10.004 (2005). 16038855 10.1016/j.math.2004.10.004

[13] Barnes, C. J., Van Steyn, S. J. & Fischer, R. A. The effects of age, sex, and shoulder dominance on range of motion of the shoulder. *Journal of Shoulder and Elbow Surgery* **10**, 242–246, 10.1067/mse.2001.115270 (2001). 11408905 10.1067/mse.2001.115270

[14] Kuhlman, K. A. Cervical range of motion in the elderly. *Archives of Physical Medicine and Rehabilitation* **74**, 1071–1079, 10.1016/0003-9993(93)90064-H (1993). 8215859 10.1016/0003-9993(93)90064-h

[15] Martin, K. L. *et al*. Cognitive Function, Gait, and Gait Variability in Older People: A Population-Based Study. *The Journals of Gerontology: Series A* **68**, 726–732, 10.1093/gerona/gls224 (2013). 10.1093/gerona/gls224 23112113

[16] Heiderscheit, B. C. Movement Variability as a Clinical Measure for Locomotion. *Journal of Applied Biomechanics* **16**, 419–427, 10.1123/jab.16.4.419 (2000).

[17] Haar, S., Donchin, O. & Dinstein, I. Individual Movement Variability Magnitudes Are Explained by Cortical Neural Variability. *J. Neurosci.* **37**, 9076–9085, 10.1523/JNEUROSCI.1650-17.2017 (2017). 28821678 10.1523/JNEUROSCI.1650-17.2017 PMC6596801

[18] Park, C. *et al*. Toward Remote Assessment of Physical Frailty Using Sensor-based Sit-to-stand Test. *Journal of Surgical Research* **263**, 130–139, 10.1016/j.jss.2021.01.023 (2021). 33652175 10.1016/j.jss.2021.01.023 PMC9113630

[19] Cellina, M. *et al*. Digital Twins: The New Frontier for Personalized Medicine? *Applied Sciences* **13**, 7940, 10.3390/app13137940 (2023).

[20] Vallée, A. Digital twin for healthcare systems. *Front. Digit. Health* **5**, 1253050, 10.3389/fdgth.2023.1253050 (2023). 37744683 10.3389/fdgth.2023.1253050 PMC10513171

[21] Konstantinidis, E. I. *et al*. Design, Implementation, and Wide Pilot Deployment of FitForAll: An Easy to use Exergaming Platform Improving Physical Fitness and Life Quality of Senior Citizens. *IEEE J. Biomed. Health Inform.* **20**, 189–200, 10.1109/JBHI.2014.2378814 (2016). 26731797 10.1109/JBHI.2014.2378814

[22] Goumopoulos, C., Drakakis, E. & Gklavakis, D. Feasibility and Acceptance of Augmented and Virtual Reality Exergames to Train Motor and Cognitive Skills of Elderly. *Computers* **12**, 52, 10.3390/computers12030052 (2023).

[23] Adcock, M., Sonder, F., Schättin, A., Gennaro, F. & de Bruin, E. D. A usability study of a multicomponent video game-based training for older adults. *Eur. Rev. Aging Phys. Act.* **17**, 3, 10.1186/s11556-019-0233-2 (2020). 31938075 10.1186/s11556-019-0233-2 PMC6955093

[24] Olugbade, T. *et al*. Human Movement Datasets: An Interdisciplinary Scoping Review. *ACM Computing Surveys* **55**, 1–29, 10.1145/3534970 (2023).

[25] Mandery, C., Terlemez, O., Do, M., Vahrenkamp, N. & Asfour, T. The KIT whole-body human motion database. In *2015 International Conference on Advanced Robotics (ICAR)*, 329–336, 10.1109/ICAR.2015.7251476 (IEEE, Istanbul, Turkey, 2015).

[26] Liang, P. *et al*. An Asian-centric human movement database capturing activities of daily living. *Scientific Data* **7**, 290, 10.1038/s41597-020-00627-7 (2020). 32901007 10.1038/s41597-020-00627-7 PMC7479610

[27] Ni, B., Wang, G. & Moulin, P. RGBD-HuDaAct: A color-depth video database for human daily activity recognition. In *2011 IEEE International Conference on Computer Vision Workshops (ICCV Workshops)*, 1147–1153, 10.1109/ICCVW.2011.6130379 (IEEE, Barcelona, Spain, 2011).

[28] Tonkin, E. L. *et al*. A multi-sensor dataset with annotated activities of daily living recorded in a residential setting. *Sci Data* **10**, 162, 10.1038/s41597-023-02017-1 (2023). 36959280 10.1038/s41597-023-02017-1 PMC10036321

[29] Dong, A. *et al*. A new kinematic dataset of lower limbs action for balance testing. *Sci Data* **10**, 209, 10.1038/s41597-023-02105-2 (2023). 37059747 10.1038/s41597-023-02105-2 PMC10104813

[30] Leightley, D., Yap, M. H., Coulson, J., Barnouin, Y. & McPhee, J. S. Benchmarking human motion analysis using kinect one: An open source dataset. In *2015 Asia-Pacific Signal and Information Processing Association Annual Summit and Conference (APSIPA)*, 1–7 (IEEE, 2015).

[31] García-de-Villa, S., Jiménez-Martín, A. & García-Domínguez, J. J. A database of physical therapy exercises with variability of execution collected by wearable sensors. *Sci Data* **9**, 266, 10.1016/10.1038/s41597-022-01387-2 (2022). 35661743 10.1038/s41597-022-01387-2 PMC9166805

[32] Grouvel, G., Carcreff, L., Moissenet, F. & Armand, S. A dataset of asymptomatic human gait and movements obtained from markers, IMUs, insoles and force plates. *Sci Data* **10**, 180, 10.1038/s41597-023-02077-3 (2023). 36997555 10.1038/s41597-023-02077-3 PMC10063557

[33] Hanisch, S., Pogrzeba, L., Muschter, E., Li, S.-C. & Strufe, T. A kinematic dataset of locomotion with gait and sit-to-stand movements of young adults. *Scientific Data* **11**, 1209, 10.1038/s41597-024-04020-6 (2024). 39521807 10.1038/s41597-024-04020-6 PMC11550319

[34] Losing, V. & Hasenjäger, M. A Multi-Modal Gait Database of Natural Everyday-Walk in an Urban Environment. *Sci Data* **9**, 473, 10.1038/s41597-022-01580-3 (2022). 35922448 10.1038/s41597-022-01580-3 PMC9349224

[35] Mehdizadeh, S. *et al*. The Toronto older adults gait archive: Video and 3D inertial motion capture data of older adults’ walking. *Sci Data* **9**, 398, 10.1038/s41597-022-01495-z (2022). 35817777 10.1038/s41597-022-01495-z PMC9272879

[36] Santos, G., Wanderley, M., Tavares, T. & Rocha, A. A multi-sensor human gait dataset captured through an optical system and inertial measurement units. *Sci Data* **9**, 545, 10.1038/s41597-022-01638-2 (2022). 36071060 10.1038/s41597-022-01638-2 PMC9452504

[37] Van Der Zee, T. J., Mundinger, E. M. & Kuo, A. D. A biomechanics dataset of healthy human walking at various speeds, step lengths and step widths. *Sci Data* **9**, 704, 10.1038/s41597-022-01817-1 (2022). 36385009 10.1038/s41597-022-01817-1 PMC9669008

[38] Vögele, A., Krüger, B. & Klein, R. Efficient Unsupervised Temporal Segmentation of Human Motion. In *Eurographics/ ACM SIGGRAPH Symposium on Computer Animation*, 15 (2014).

[39] Lin, J. F.-S., Karg, M. & Kulic, D. Movement Primitive Segmentation for Human Motion Modeling: A Framework for Analysis. *IEEE Trans. Human-Mach. Syst.* **46**, 325–339, 10.1109/THMS.2015.2493536 (2016).

[40] Sebernegg, A., Kán, P. & Kaufmann, H. Motion Similarity Modeling – A State of the Art Report. *arXiv:2008.05872 [cs.GR]*, 10.48550/arXiv.2008.05872 (2020).

[41] Zunino, A., Cavazza, J. & Murino, V. Revisiting Human Action Recognition: Personalization vs. Generalization. *arXiv:1605.00392 [cs.CV]*, 10.48550/arXiv.1605.00392 (2016).

[42] Keates, S., Clarkson, P. J., Harrison, L.-A. & Robinson, P. Towards a practical inclusive design approach. In *Proceedings on the 2000 Conference on Universal Usability - CUU ’00*, 45–52, 10.1145/355460.355471 (ACM Press, Arlington, Virginia, United States, 2000).

[43] Newell, A. Older people as a focus for inclusive design. *Gerontechnology* **4**, 190–199, 10.4017/gt.2006.04.04.003.00 (2006).

[44] Li, S.-C. & Fitzek, F. H. P. Digitally embodied lifespan neurocognitive development and Tactile Internet: Transdisciplinary challenges and opportunities. *Frontiers in Human Neuroscience* **17**, 1116501, 10.3389/fnhum.2023.1116501 (2023). 36845878 10.3389/fnhum.2023.1116501 PMC9950571

[45] Sin, J., L. Franz, R., Munteanu, C. & Barbosa Neves, B. Digital Design Marginalization: New Perspectives on Designing Inclusive Interfaces. In *Proceedings of the 2021 CHI Conference on Human Factors in Computing Systems*, 1–11, 10.1145/3411764.3445180 (ACM, Yokohama Japan, 2021).

[46] Das, S. *et al*. Toyota Smarthome: Real-World Activities of Daily Living. In *IEEE Int. Conf. Computer Vision*, 833-842, 10.1109/ICCV.2019.00092 (2019).

[47] Jang, J. *et al*. ETRI-Activity3D: A Large-Scale RGB-D Dataset for Robots to Recognize Daily Activities of the Elderly. In *2020 IEEE/RSJ International Conference on Intelligent Robots and Systems (IROS)*, 10990–10997, 10.1109/IROS45743.2020.9341160 (IEEE, Las Vegas, NV, USA, 2020).

[48] Linkenauger, S. A., Witt, J. K., Stefanucci, J. K., Bakdash, J. Z. & Proffitt, D. R. The effects of handedness and reachability on perceived distance. *Journal of Experimental Psychology: Human Perception and Performance* **35**, 1649–1660, 10.1037/a0016875 (2009). 19968426 10.1037/a0016875 PMC3291021

[49] Rosenbaum, D. A. Reaching while walking: Reaching distance costs more than walking distance. *Psychonomic Bulletin & Review* **15**, 1100–1104, 10.3758/PBR.15.6.1100 (2008). 19001574 10.3758/PBR.15.6.1100

[50] Srinivasan, D. & Martin, B. Object and target size interactions in placement tasks. *Proceedings of the Human Factors and Ergonomics Society Annual Meeting* **52**, 940–944, 10.1177/154193120805201309 (2008).

[51] Gulletta, G. *et al*. A Human-like Upper-limb Motion Planner: Generating naturalistic movements for humanoid robots. *International Journal of Advanced Robotic Systems* **18**(2), 1–31, 10.1177/1729881421998585 (2021).

[52] Odesanmi, G. A., Wang, Q. & Mai, J. Skill learning framework for human–robot interaction and manipulation tasks. *Robotics and Computer-Integrated Manufacturing* **79**, 1–10, 10.1016/j.rcim.2022.102444 (2023).

[53] Zhou, L., Li, W., Ogunbona, P. & Zhang, Z. Semantic action recognition by learning a pose lexicon. *Pattern Recognition* **72**, 548–562, 10.1016/j.patcog.2017.06.035 (2017).

[54] Waugh, J. L. S. *et al*. Online Learning of Gait Models From Older Adult Data. *IEEE Trans. Neural Syst. Rehabil. Eng.* **27**, 733–742, 10.1109/TNSRE.2019.2904477 (2019). 30872234 10.1109/TNSRE.2019.2904477

[55] Connor, P. & Ross, A. Biometric recognition by gait: A survey of modalities and features. *Computer Vision and Image Understanding* **167**, 1–27, 10.1016/j.cviu.2018.01.007 (2018).

[56] Liebers, J. *et al*. Kinetic Signatures: A Systematic Investigation of Movement-Based User Identification in Virtual Reality. In *Proceedings of the CHI Conference on Human Factors in Computing Systems*, 1–19, 10.1145/3613904.3642471 (ACM, Honolulu HI USA, 2024).

[57] Hanisch, S., Muschter, E., Hatzipanayioti, A., Li, S.-C. & Strufe, T. Understanding Person Identification Through Gait. *PoPETs* **2023**, 177–189, 10.56553/popets-2023-0011 (2023).

[58] Arac, A. Machine Learning for 3D Kinematic Analysis of Movements in Neurorehabilitation. *Curr Neurol Neurosci Rep* **20**, 29, 10.1007/s11910-020-01049-z (2020). 32542455 10.1007/s11910-020-01049-z PMC7397814

[59] Rybnikár, F., Kačerová, I., Hořejší, P. & Šimon, M. Ergonomics Evaluation Using Motion Capture Technology—Literature Review. *Applied Sciences* **13**, 162, 10.3390/app13010162 (2022).

[60] Ashcroft, R. E. The declaration of Helsinki. In Ezekiel J. Emanuel, *The Oxford textbook of clinical research ethics*. New York: Oxford University Press, 141–148 (2008).

[61] Harris, P. A. *et al*. Research electronic data capture (REDCap)—A metadata-driven methodology and workflow process for providing translational research informatics support. *Journal of Biomedical Informatics* **42**, 377–381, 10.1016/j.jbi.2008.08.010 (2009). 18929686 10.1016/j.jbi.2008.08.010 PMC2700030

[62] Harris, P. A. *et al*. The REDCap consortium: Building an international community of software platform partners. *Journal of Biomedical Informatics* **95**, 1–24, 10.1016/j.jbi.2019.103208 (2019). 10.1016/j.jbi.2019.103208 PMC7254481 31078660

[63] Kuorinka, I. *et al*. Standardised Nordic questionnaires for the analysis of musculoskeletal symptoms. *Applied Ergonomics* **18**, 233–237 (1987). 15676628 10.1016/0003-6870(87)90010-x

[64] Caffier, G., Steinberg, U. & Liebers, F. Praxisorientiertes Methodeninventar zur Belastungs- und Beanspruchungsbeurteilung im Zusammenhang mit arbeitsbedingten Muskel-Skelett-Erkrankungen. Tech. Rep. Fb850, Bundesanstalt für Arbeitsschutz und Arbeitsmedizin, Dortmund/Berlin (1999).

[65] Oldfield, R. The assessment and analysis of handedness: The Edinburgh inventory. *Neuropsychologia* **9**, 97–113, 10.1016/0028-3932(71)90067-4 (1971). 5146491 10.1016/0028-3932(71)90067-4

[66] Caeiro-Rodríguez, M., Otero-González, I., Mikic-Fonte, F. A. & Llamas-Nistal, M. A Systematic Review of Commercial Smart Gloves: Current Status and Applications. *Sensors* **21**, 1–31, 10.3390/s21082667 (2021). 10.3390/s21082667 PMC8070066 33920101

[67] Bosch Sensortec. BMI160 - Small, low power inertial measurement unit. Data Sheet BST-BMI160-DS000-09, Bosch https://www.bosch-sensortec.com/media/boschsensortec/downloads/datasheets/bst-bmi160-ds000.pdf (2020).

[68] Schall, M. C., Chen, H. & Cavuoto, L. Wearable inertial sensors for objective kinematic assessments: A brief overview. *Journal of Occupational and Environmental Hygiene* **19**, 501–508, 10.1080/15459624.2022.2100407 (2022). 35853137 10.1080/15459624.2022.2100407

[69] Bergamini, E. *et al*. Estimating Orientation Using Magnetic and Inertial Sensors and Different Sensor Fusion Approaches: Accuracy Assessment in Manual and Locomotion Tasks. *Sensors* **14**, 18625–18649, 10.3390/s141018625 (2014). 25302810 10.3390/s141018625 PMC4239903

[70] Cereatti, A. *et al*. ISB recommendations on the definition, estimation, and reporting of joint kinematics in human motion analysis applications using wearable inertial measurement technology. *Journal of Biomechanics* **173**, 1–11, 10.1016/j.jbiomech.2024.112225 (2024). 10.1016/j.jbiomech.2024.112225 39032224

[71] Hou, X., Lu, Y. & Dey, S. Wireless VR/AR with Edge/Cloud Computing. In *2017 26th International Conference on Computer Communication and Networks (ICCCN)*, 10.1109/ICCCN.2017.8038375 (2017).

[72] Mihcin, S., Ciklacandir, S., Kocak, M. & Tosun, A. Wearable Motion Capture System Evaluation for Biomechanical Studies for Hip Joints. *J. Biomech. Eng.* **143**(4), 044504-01–044504-08, 10.1115/1.4049199 (2021). 34043760 10.1115/1.4049199

[73] Mihcin, S. Simultaneous validation of wearable motion capture system for lower body applications: Over single plane range of motion (ROM) and gait activities. *Biomedical Engineering / Biomedizinische Technik* **67**, 185–199, 10.1515/bmt-2021-0429 (2022). 35575784 10.1515/bmt-2021-0429

[74] Ciklacandir, S., Ozkan, S. & Isler, Y. A Comparison of the Performances of Video-Based and IMU Sensor-Based Motion Capture Systems on Joint Angles. In *2022 Innovations in Intelligent Systems and Applications Conference (ASYU)*, 1–5, 10.1109/ASYU56188.2022.9925507 (IEEE, Antalya, Turkey, 2022).

[75] Bohannon, R. W., Bubela, D. J., Magasi, S. R., Wang, Y.-C. & Gershon, R. C. Sit-to-stand test: Performance and determinants across the age-span. *IES* **18**, 235–240, 10.3233/IES-2010-0389 (2010). 25598584 10.3233/IES-2010-0389 PMC4293702

[76] Ryf, Chr & Weymann, A. The neutral zero method — A principle of measuring joint function. *Injury* **26**, 1–11, 10.1016/0020-1383(95)90116-7 (1995).

[77] Thompson, N. E., Demes, B., O’Neill, M. C., Holowka, N. B. & Larson, S. G. Surprising trunk rotational capabilities in chimpanzees and implications for bipedal walking proficiency in early hominins. *Nat Commun* **6**(1), 1–7, 10.1038/ncomms9416 (2015). 10.1038/ncomms9416 PMC4600717 26441046

[78] Van Criekinge, T., Hallemans, A., Van De Walle, P. & Sloot, L. H. Age- and sex-related differences in trunk kinematics during walking in able-bodied adults. *GeroScience* **46**, 2545–2559, 10.1007/s11357-023-01028-5 (2023). 38032420 10.1007/s11357-023-01028-5 PMC10828227

[79] Sung, P. S., Lee, K.-J. & Park, W.-H. Coordination of trunk and pelvis in young and elderly individuals during axial trunk rotation. *Gait & Posture* **36**, 330–331, 10.1016/j.gaitpost.2012.03.009 (2012). 22465703 10.1016/j.gaitpost.2012.03.009

[80] Oosterwijk, A., Nieuwenhuis, M., Van Der Schans, C. & Mouton, L. Shoulder and elbow range of motion for the performance of activities of daily living: A systematic review. *Physiotherapy Theory and Practice* **34**, 505–528, 10.1080/09593985.2017.1422206 (2018). 29377745 10.1080/09593985.2017.1422206

[81] Grauman, K. *et al*. Ego4D: Around the World in 3,000 Hours of Egocentric Video, *arXiv:2110.07058 [cs.CV]*, 10.48550/ARXIV.2110.07058 (2022).

[82] Subash, T. *et al*. Comparing algorithms for assessing upper limb use with inertial measurement units. *Front. Physiol.* **13**, 1–13, 10.3389/fphys.2022.1023589 (2022). 10.3389/fphys.2022.1023589 PMC9806112 36601345

[83] Beauchet, O. *et al*. Guidelines for Assessment of Gait and Reference Values for Spatiotemporal Gait Parameters in Older Adults: The Biomathics and Canadian Gait Consortiums Initiative. *Front. Hum. Neurosci.* **11**, 1–13, 10.3389/fnhum.2017.00353 (2017). 28824393 10.3389/fnhum.2017.00353 PMC5540886

[84] Mc Ardle, R. *et al*. Characterizing Walking Behaviors in Aged Residential Care Using Accelerometry, With Comparison Across Care Levels, Cognitive Status, and Physical Function: Cross-Sectional Study. *JMIR Aging* **7**, 1–14, 10.2196/53020 (2024). 10.2196/53020 PMC11185191 38842168

[85] Kiprijanovska, I., Gjoreski, H. & Gams, M. Detection of Gait Abnormalities for Fall Risk Assessment Using Wrist-Worn Inertial Sensors and Deep Learning. *Sensors* **20**, 1–21, 10.3390/s20185373 (2020). 10.3390/s20185373 PMC7571106 32961750

[86] Taylor, L. *et al*. Evaluating the effects of an exercise program (Staying UpRight) for older adults in long-term care on rates of falls: Study protocol for a randomised controlled trial. *Trials* **21**, 46, 10.1186/s13063-019-3949-4 (2020). 31915043 10.1186/s13063-019-3949-4 PMC6950827

[87] Lusardi, M. M. *et al*. Determining Risk of Falls in Community Dwelling Older Adults: A Systematic Review and Meta-analysis Using Posttest Probability. *Journal of Geriatric Physical Therapy* **40**, 1–36, 10.1519/JPT.0000000000000099 (2017). 27537070 10.1519/JPT.0000000000000099 PMC5158094

[88] O’Brien, M. K. *et al*. Augmenting Clinical Outcome Measures of Gait and Balance with a Single Inertial Sensor in Age-Ranged Healthy Adults. *Sensors* **19**(20), 4537, 1–28, 10.3390/s19204537 (2019). 10.3390/s19204537 PMC6832985 31635375

[89] Schumann, P. *et al*. Using machine learning algorithms to detect fear of falling in people with multiple sclerosis in standardized gait analysis. *Multiple Sclerosis and Related Disorders* **88**, 105721, 1–7, 10.1016/j.msard.2024.105721 (2024). 10.1016/j.msard.2024.105721 38885599

[90] Rokoko Electronics. Actor Profile. https://support.rokoko.com/hc/en-us/articles/4410415403025-Actor-Profile.

[91] Pujades, S. *et al*. The Virtual Caliper: Rapid Creation of Metrically Accurate Avatars from 3D Measurements. *IEEE Trans. Visual. Comput. Graphics* **25**, 1887–1897, 10.1109/TVCG.2019.2898748 (2019). 10.1109/TVCG.2019.2898748 30794512

[92] Loper, M., Mahmood, N., Romero, J., Pons-Moll, G. & Black, M. J. SMPL: A skinned multi-person linear model. *ACM Trans. Graph.* **34**, 1–16, 10.1145/2816795.2818013 (2015).

[93] Goodhew, S. C. & Edwards, M. Translating experimental paradigms into individual-differences research: Contributions, challenges, and practical recommendations. *Consciousness and Cognition* **69**, 14–25, 10.1016/j.concog.2019.01.008 (2019). 30685513 10.1016/j.concog.2019.01.008

[94] Bachmann, E., Yun, X. & Peterson, C. An investigation of the effects of magnetic variations on inertial/magnetic orientation sensors. In *IEEE International Conference on Robotics and Automation, 2004. Proceedings. ICRA ’04. 2004*, 1115–1122 Vol.2, 10.1109/ROBOT.2004.1307974 (IEEE, New Orleans, LA, USA, 2004).

[95] De Vries, W., Veeger, H., Baten, C. & Van Der Helm, F. Magnetic distortion in motion labs, implications for validating inertial magnetic sensors. *Gait & Posture* **29**, 535–541, 10.1016/j.gaitpost.2008.12.004 (2009). 19150239 10.1016/j.gaitpost.2008.12.004

[96] Catelani, M. *et al*. Reliability and Functional Analysis of IMU systems under temperature-based stress tests. In *2021 IEEE Int. Instrum. Meas. Technol. Conf. I2MTC*, 1–6, 10.1109/I2MTC50364.2021.9459831 (IEEE, Glasgow, United Kingdom, 2021).

[97] Capriglione, D. *et al*. Analysis of MEMS devices under temperature stress test. In *2021 IEEE 8th Int. Workshop Metrol. Aerosp. MetroAeroSpace*, 63–68, 10.1109/MetroAeroSpace51421.2021.9511744 (IEEE, Naples, Italy, 2021).

[98] Rokoko Electronics. Rokoko studio guides: Filters explained. https://support.rokoko.com/hc/en-us/articles/4410415474321-Filters-explained.

[99] Pogrzeba, L. *et al*. The CeTI-Age-Kinematics Dataset: Fully-body kinematic IMU Data of the Elderly and Young Adults in Daily Tasks. *Figshare*10.6084/m9.figshare.26983645 (2024).

[100] Jeung, S. *et al*. Motion-BIDS: An extension to the brain imaging data structure to organize motion data for reproducible research. *Scientific Data* **11**, 716, 10.1038/s4159‐7‐024‐03559‐8 (2024). 38956071 PMC11219788

[101] Gorgolewski, K. J. *et al*. The brain imaging data structure, a format for organizing and describing outputs of neuroimaging experiments. *Scientific Data* **3**, 160044, 1–9, 10.1038/sdata.2016.44 (2016). 10.1038/sdata.2016.44 PMC4978148 27326542

[102] Oliver-Taylor, A. *et al*. Bids-specification. *Zenodo*, 10.5281/zenodo.10175846 (2023).

[103] Dai, H., Cai, B., Song, J. & Zhang, D. Skeletal Animation Based on BVH Motion Data. In *2010 2nd Int. Conf. Inf. Eng. Comput. Sci*., 1–4, 10.1109/ICIECS.2010.5678292 (IEEE, Wuhan, China, 2010).

[104] Scataglini, S. & Truijen, S. Overview of software and file exchange formats in 3D and 4D body shape scanning. In *Proc. 7th Int. Digit. Hum. Model. Symp.* **7**(1), 11, 1–9, 10.17077/dhm.31757 (University of Iowa Libraries Publishing, 2022).

[105] Grood, E. S. & Suntay, W. J. A Joint Coordinate System for the Clinical Description of Three-Dimensional Motions: Application to the Knee. *Journal of Biomechanical Engineering* **105**, 136–144, 10.1115/1.3138397 (1983). 6865355 10.1115/1.3138397

[106] Wu, G. *et al*. ISB recommendation on definitions of joint coordinate system of various joints for the reporting of human joint motion—part I: Ankle, hip, and spine. *Journal of Biomechanics* **35**, 543–548, 10.1016/S0021-9290(01)00222-6 (2002). 11934426 10.1016/s0021-9290(01)00222-6

[107] Wu, G. *et al*. ISB recommendation on definitions of joint coordinate systems of various joints for the reporting of human joint motion—Part II: Shoulder, elbow, wrist and hand. *Journal of Biomechanics* **38**, 981–992, 10.1016/j.jbiomech.2004.05.042 (2005). 15844264 10.1016/j.jbiomech.2004.05.042

[108] Autodesk Maya. Maya Help ∣ HumanIK character structure ∣ Autodesk. https://help.autodesk.com/view/MAYAUL/2024/ENU/?guid=GUID-5DEFC6E5-033C-45D5-9A0E-224E7A35131B (2024).

[109] Autodesk MotionBuilder Software. https://www.autodesk.com/products/motionbuilder/overview.

[110] Wechsler, I. *et al*. Method for Using IMU-Based Experimental Motion Data in BVH Format for Musculoskeletal Simulations via OpenSim. *Sensors* **23**, 5423, 10.3390/s23125423 (2023). 37420590 10.3390/s23125423 PMC10303752

[111] Ruiz-Ruiz, L., Jimenez, A. R., Garcia-Villamil, G. & Seco, F. Detecting Fall Risk and Frailty in Elders with Inertial Motion Sensors: A Survey of Significant Gait Parameters. *Sensors* **21**, 6918, 10.3390/s21206918 (2021). 34696131 10.3390/s21206918 PMC8538337

[112] Ambike, S. & Schmiedeler, J. P. Invariant geometric characteristics of spatial arm motion. *Exp Brain Res* **229**, 113–124, 10.1007/s00221-013-3599-9 (2013). 23771586 10.1007/s00221-013-3599-9

[113] Klein Breteler, M. D., Meulenbroek, R. G. & Gielen, S. C. Geometric features of workspace and joint-space paths of 3d reaching movements. *Acta Psychologica* **100**, 37–53, 10.1016/S0001-6918(98)00024-9 (1998). 9844555 10.1016/s0001-6918(98)00024-9

[114] Ponsiglione, A. M. *et al*. Statistical Analysis and Kinematic Assessment of Upper Limb Reaching Task in Parkinson’s Disease. *Sensors* **22**, 1708, 10.3390/s22051708 (2022). 35270853 10.3390/s22051708 PMC8915106

[115] Gates, D. H., Walters, L. S., Cowley, J., Wilken, J. M. & Resnik, L. Range of Motion Requirements for Upper-Limb Activities of Daily Living. *Am. J. Occup. Ther.* **70**, 7001350010p1–7001350010p10, 10.5014/ajot.2016.015487 (2016). 26709433 10.5014/ajot.2016.015487 PMC4690598

[116] Abend, W., Bizzi, E. & Morasso, P. Human Arm Trajectory Formation. *Brain* **105**, 331–348, 10.1093/brain/105.2.331 (1982). 7082993 10.1093/brain/105.2.331

[117] Li, Z., Milutinović, D. & Rosen, J. From reaching to reach-to-grasp: The arm posture difference and its implications on human motion control strategy. *Exp Brain Res* **235**, 1627–1642, 10.1007/s00221-017-4890-y (2017). 28265688 10.1007/s00221-017-4890-y

[118] Chaffin, D. B., Faraway, J. J., Zhang, X. & Woolley, C. Stature, Age, and Gender Effects on Reach Motion Postures. *Hum Factors* **42**, 408–420, 10.1518/001872000779698222 (2000). 11132802 10.1518/001872000779698222

[119] Nordin, N., Xie, S. & Wünsche, B. Assessment of movement quality in robot- assisted upper limb rehabilitation after stroke: A review. *J NeuroEngineering Rehabil* **11**, 137, 10.1186/1743-0003-11-137 (2014). 10.1186/1743-0003-11-137 PMC4180322 25217124

[120] Albrecht, S. *et al*. Imitating human reaching motions using physically inspired optimization principles. In *2011 11th IEEE-RAS Int. Conf. Humanoid Robots*, 602–607, 10.1109/Humanoids.2011.6100856 (IEEE, Bled, Slovenia, 2011).

[121] Lin, J. F.-S., Joukov, V. & Kulić, D. Classification-based Segmentation for Rehabilitation Exercise Monitoring. *Journal of Rehabilitation and Assistive Technologies Engineering* **5**, 1–12, 10.1177/2055668318761523 (2018). 10.1177/2055668318761523 PMC6453256 31191926

[122] López-Nava, I. H., Arnrich, B., Muñoz-Meléndez, A. & Güneysu, A. Variability Analysis of Therapeutic Movements using Wearable Inertial Sensors. *J Med Syst* **41**, 7, 10.1007/s10916-016-0645-8 (2017). 27848176 10.1007/s10916-016-0645-8

[123] Vandenberghe, A., Levin, O., De Schutter, J., Swinnen, S. & Jonkers, I. Three-dimensional reaching tasks: Effect of reaching height and width on upper limb kinematics and muscle activity. *Gait & Posture* **32**, 500–507, 10.1016/j.gaitpost.2010.07.009 (2010). 20729085 10.1016/j.gaitpost.2010.07.009

[124] Bohannon, R. W., Myers, B. J., Tudini, F. T., Clark, J. T. & Manor, J. P. Kinematics of shoulder, trunk, pelvis, and hip while reaching forward to progressively distant targets. *Journal of Bodywork and Movement Therapies* **24**, 221–226, 10.1016/j.jbmt.2020.03.003 (2020). 32825992 10.1016/j.jbmt.2020.03.003

[125] Faraway, J. J., Reed, M. P. & Wang, J. Modelling Three-Dimensional Trajectories by Using Bézier Curves with Application to Hand Motion. *J. R. Stat. Soc. Ser. C Appl. Stat.* **56**, 571–585, 10.1111/j.1467-9876.2007.00592.x (2007).

[126] Yang, N., Zhang, M., Huang, C. & Jin, D. Synergic analysis of upper limb target-reaching movements. *Journal of Biomechanics* **35**, 739–746, 10.1016/S0021-9290(02)00018-0 (2002). 12020993 10.1016/s0021-9290(02)00018-0

[127] Bockemühl, T., Troje, N. F. & Dürr, V. Inter-joint coupling and joint angle synergies of human catching movements. *Human Movement Science* **29**, 73–93, 10.1016/j.humov.2009.03.003 (2010). 19945187 10.1016/j.humov.2009.03.003

[128] Yarkoni, T. *et al*. PyBIDS: Python tools for BIDS datasets. *JOSS* **4**, 1294, 10.21105/joss.01294 (2019). 32775955 10.21105/joss.01294 PMC7409983

[129] Yarkoni, T. *et al*. PyBIDS: Python tools for BIDS datasets, *Zenodo*, 10.5281/ZENODO.11244297 (2024).

[130] Holden, D. BVHView https://github.com/orangeduck/BVHView (2023).

[131] Autodesk. FBX Review Cross-platform 3D model viewer (2022). Version 1.5.3.0, https://www.autodesk.com/products/fbx/fbx-review.

[132] Thillet, L. BVH Animation BETA http://lo-th.github.io/olympe/ (2014).

[133] Smith, H. J., Zheng, Q., Li, Y., Jain, S. & Hodgins, J. K. A Method for Animating Children’s Drawings of the Human Figure. *ACM Trans. Graph.* **42**, 1–15, 10.1145/3592788 (2023).

[134] Karuzaki, E. *et al*. Realistic Virtual Humans for Cultural Heritage Applications. *Heritage* **4**, 4148–4171, 10.3390/heritage4040228 (2021).

[135] Reitz, K. & Schlusser, T. Shipping great code. In *The Hitchhiker’s Guide to Python*, chap. 6, 167–182 (O’Reilly Media, Sebastopol, CA, 2016), 1st edn.

[136] The Bioconda Team. *et al*. Bioconda: Sustainable and comprehensive software distribution for the life sciences. *Nat Methods* **15**, 475–476, 10.1038/s41592-018-0046-7 (2018). 29967506 10.1038/s41592-018-0046-7 PMC11070151

