# Supplementary material for: A Full-Body IMU-Based Motion Dataset of Daily Tasks by Older and Younger Adults
Source: Sci Data. 2025 Mar 29;12:531. doi: 10.1038/s41597-025-04818-y (PMC11954993; doi:10.1038/s41597-025-04818-y)
Supplement: Supplementary file 1 — Supplementary: A Full-Body IMU-Based Motion Dataset of Daily Tasks by Older and Younger Adults [file 41597_2025_4818_MOESM1_ESM.pdf]

Supplementary: A Full-Body IMU-Based Motion Dataset of Daily Tasks by Older and Younger Adults

Contents

1 Basic demographic and anthropometric data by participant 2

2 Design considerations for the selected movement tasks 3

3 Acquisition setups and procedures 4

4 Additional information on data records 7

5 BVH bone labels and hierarchy 9

6 Column field names in the `participants.tsv` file 10

7 Additional validation approaches 12

8 Segmentation 14

References 16

1 Basic demographic and anthropometric data by participant

participant_id	participant_group	sex	age	handedness	mass	bmi	a01	a02	a05
sub-d02	OA	m	66	right	95.10	30.0	178.1	168.1	149.5
sub-d04	OA	f	68	right	61.65	23.2	163.1	151.5	137.8
sub-d05	OA	m	70	right	80.90	24.4	182.1	171.6	150.5
sub-d06	OA	m	73	right	83.25	29.1	169.1	159.1	144.0
sub-d07	OA	m	75	right	88.70	31.7	167.4	158.2	146.4
sub-d09	OA	m	66	right	106.45	35.9	172.1	162.6	144.9
sub-d11	OA	f	73	right	58.15	22.3	161.6	151.1	132.6
sub-d13	OA	m	73	right	92.40	30.4	174.4	166.1	145.0
sub-d14	OA	f	70	right	74.15	27.9	163.1	150.6	138.0
sub-d15	OA	m	68	right	80.60	27.3	171.8	163.3	147.0
sub-d16	OA	m	67	right	67.65	23.9	168.1	158.1	139.5
sub-d17	OA	f	67	left	65.45	25.5	160.1	150.1	140.0
sub-d18	OA	f	69	right	64.30	21.2	174.1	162.6	146.0
sub-d20	OA	m	67	right	70.00	23.3	173.2	163.1	144.0
sub-d21	OA	f	68	right	55.85	23.2	155.1	144.3	128.0
sub-d22	OA	f	71	right	58.55	23.1	159.1	147.6	135.0
sub-d23	OA	f	72	right	86.00	32.7	162.1	151.1	134.0
sub-d25	OA	m	71	right	70.20	22.8	175.6	166.1	148.5
sub-d26	OA	f	69	left	63.95	23.5	165.1	156.6	140.0
sub-d27	YA	m	28	right	80.05	24.4	181.1	168.1	148.8
sub-d28	YA	f	19	right	51.90	18.7	166.6	154.6	137.0
sub-d29	YA	f	27	right	66.10	25.8	160.1	149.6	132.4
sub-d30	YA	f	19	right	61.00	19.8	175.6	165.1	146.8
sub-d31	YA	m	24	right	63.40	20.0	178.1	165.6	146.3
sub-d32	YA	f	25	right	49.60	18.6	163.1	151.2	134.9
sub-d33	YA	f	21	right	63.00	22.8	166.3	154.6	138.0
sub-d34	YA	m	20	right	74.40	21.8	184.6	174.1	155.0
sub-d35	YA	m	22	right	64.85	20.4	178.1	167.1	148.0
sub-d36	YA	m	21	right	91.65	27.3	183.1	174.1	152.5
sub-d37	OA	f	73	right	67.35	26.1	160.6	148.1	134.5
sub-d38	YA	f	20	right	65.85	21.5	174.9	163.0	147.3
sub-d39	YA	f	22	right	69.40	24.7	167.7	157.2	140.1

Table S1. Overview of basic demographic and anthropometric data, sorted in ascending order by participant IDs (see column `participant_id`). The column `participant_group` denotes older adults (OA) and younger adults (YA), respectively; `sex` refers to male (m) and female (f). `a01` states the body height (see Tab. S2(a), 1), `a02` the eye level (Tab. S2(a), 2), and `a05` the height of the right shoulder (see Tab. S2(a), 5).

Other than the basic demographic and anthropometric data shown in Tab. S1, the participants were asked to report any discomfort or limitations that might impact their ability to perform daily movements. This included the following conditions (cf. column field names `[5:28)`, denoting extended demographic data in Tab. S5): gout, rheumatoid arthritis, severe osteoporosis, significant foot deformities, polio, multiple sclerosis, Parkinson's disease, stroke, and severe scoliosis. Five participants reported impairments: sub-d02 reported issues with the right meniscus; sub-d06 indicated gout; sub-d07 mentioned arthropathy and the use of prostheses or orthoses; and sub-d16 reported arthrosis in the hip region. For further details, please refer to the source code and data provided in the *`participant.tsv`* file.

2 Design considerations for the selected movement tasks

In addition to ensuring an age-comparative sample including two age groups—older adults (66—75 years) and younger adults (19—28 years)—and recording multiple repetitions per task, we adhered to several other principles when designing the movement tasks for the *CeTI-Age-Kinematics* dataset, as outlined below:

1. **Naturalistic and unrestricted motion execution including task variations.** Algorithms trained on composite and variably executed movements are more likely to develop robust motion representations and generalize effectively to novel data or situations[1,2]. Thus, we designed the movement tasks to allow unrestricted movement across all anatomical planes, accommodating variations in execution speeds, styles, and environmental conditions including object types and positions (see boldface text in Tab. 2). Additionally, participants' movements exhibit natural variations that reflect individual and age-related differences in daily activities, such as the decrease in the range of motion (ROM) with aging[3]. By incorporating movements from both younger adults (YA) and older adults (OA), the dataset captures real-world variability in populations of different ages. This not only enhances the representativeness of the movements but also make it possible to train machine learning(ML)-algorithms on a broader spectrum of natural motion patterns.
2. **Detailed task instructions with supervised practice.** Live motion demonstrations shown by another individual (e.g., the experimenter) pose challenges in controlling data quality affecting movement coverage and correctness[4]. Additionally, motion demonstrations may influence participants' motion performances due to priming effects, such as altering timing and distribution of peak velocities during reaching[5]. Therefore, all motion tasks included in our battery were verbally communicated through read-aloud the task descriptions and reinforced with supervised practice trials. Beyond their utility during data acquisition, detailed task description can serve multiple purposes for data modeling and analyses, such as facilitating the development of ML models for action annotation and classification[6–8].
3. **Isolated and simultaneously executed movements.** Human movements typically involve simultaneous or sequential actions, with isolated actions being rare[9]. Everyday tasks consist of logical sequences of sub-actions, sometimes overlapping in time. To capture this complexity, we recorded actions in isolation (`w01-04`, `o01-02` in Tab. 2) and in combination (`o03-06` in Tab. 2).
4. **Object transports in forward and reversal directions.** Building on the previous point, interacting with objects often involves different motion phases and sub-movements such as a forward transport of the object towards a target location, followed by a subsequent transport in the reverse direction from the target back to the initial position[10,11]. Therefore, all object manipulation tasks without locomotion (see Tab. 2, `e01-05`, `g01-02`, `h01-02`, `o01-02`, `r01-08`, `u01`) encompass both approaching and receding actions of object transfer.
5. **Movement interactions with imaginary objects.** The dataset incorporates the concept of imaginary objects, which are mentally represented rather than physically present[12] (see Tab.2, `e01`, `e03`, `o02`, `o04`, `o06`). Individual and age-related differences in mental imageries are relevant in virtual reality environments[12]. By including imaginary objects, the dataset enables researchers to investigate the effects of object presence or absence on task execution[13] and gain insights into effects of mental representations of movements and their retrieval from memory on various aspects of motion execution. Previous research has shown that interactions with real, or imaginary objects can affect motion trajectories[14]. Additionally, analyzing these interactions can aid the designs of virtual environments, which may lack certain features such as the sensation of weight and external movement constraints (e. g., restricted motion of a door due to its hinges). The absence of such features can alter movement patterns in virtual reality and influence the demands on motion control in virtual reality environments.

3 Acquisition setups and procedures

Additional measures of key body and environmental features

To enhance the interpretability of the recorded kinematic data, we collected additional measures to more effectively characterize the physical features of the participants and the task environments.

1. **Anthropometric measures.** Anthropometric variables are essential for characterizing individual differences in movements, for instance, reaching different target positions in reaching tasks [15–18] or variations in gait parameters in walking tasks[19]. Thus, these measures are valuable for interpreting motion capture (MoCap) data and for creating customized avatars tailored to individuals[20]. Avatars can be used to visualize and interpret human motion data, showing detailed movement execution and posture within the surrounding space[21]. They also play a role in research on motion perception and embodiment[22,23].
2. **Spatial distance measures.** Feet positioning affects postural sway and center of pressure during quiet stance[24] and influences movement dynamics in balance tasks[25]. Similarly, factors such as object size[26], orientation[27], and distance[28] influence motion planning and execution in reaching and placement tasks. While the MoCap data suit provides relative positioning information of the person, it lacks details on the absolute object distance and orientation relative to the body, which are crucial for interpreting movements. To address this, we employed a practical measurement approach of capturing the position of the feet and anatomical landmarks at the hip and shoulder relative to the main interaction object (see Spatial setup configuration). This approach applies to tasks involving object interactions (does *not* apply for tasks labeled by TL-IDs `c01`, `s01`, `u01`, and `w01-04` in Tab. 2).

Anthropometric body measures and actor body profiles

(a) Full body height **(b) Full body breadth** **(c) Right (R) upper limb (UL)**

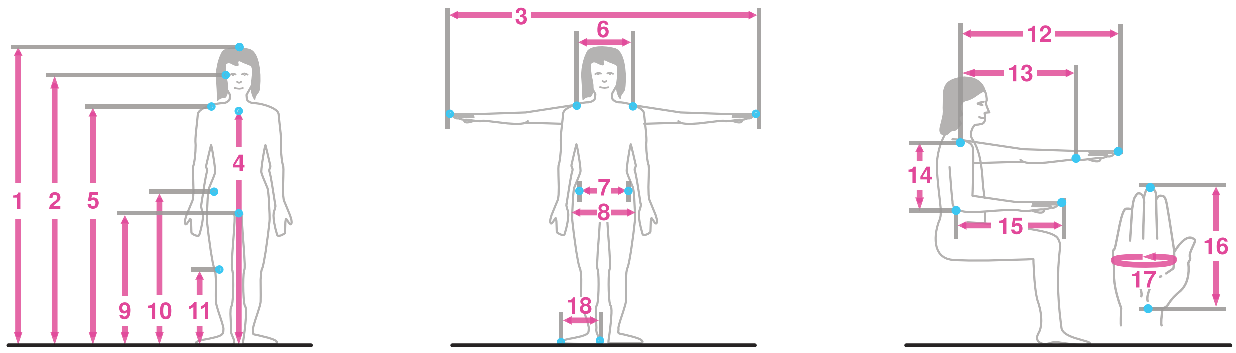

1 – body height (stature)[29–31]
2 – eye level[29,32]
4 – R shoulder height (variant A)[31]
5 – R shoulder height (variant B)[29,32]
9 – inseam (crotch) height[20,29]
10 – pelvis (iliac spine) height[29,31]
11 – R knee height[31]

3 – arm span[20,31,32]
6 – shoulder (biacromial) width[29,31,32]
7 – pelvis width[31]
8 – hip width on inseam height[20]
18 – R foot length[29,31]
17 – R hand circumference[33]

12 – R UL length (variant A)[32]
13 – R UL length (variant B)[20]
14 – R shoulder-elbow length[32]
15 – R manus length[29,31,32]
16 – R hand length[29,30,33]

Table S2. Overview of anthropometric body measures: (a) height and (b) breadth measures of the full body, and (c) measures of only the right (R) upper limb (UL).

Manual acquisition of measurements was performed using a wall-mounted stadiometer (Hilitand), standard tape measure and metric ruler, a spirit level, a sheet of paper to mark the footprint, and a scale equipped with bioelectrical impedance analysis (Tanita, model UM-076). All measurements were taken in socks, except weights, which were collected barefoot. For further information about the measurement process, please refer to the session protocols that are published as part of the dataset (see Data Records).

Measurements included overall body height (stature) (cf. Tab. S1, `a01`), eye height (cf. Tab. S1, `a02`), right shoulder height (cf. Tab. S1, `a05`), inseam, pelvis height, and knee height (see Tab. S2a). Additionally, assessments of shoulder, pelvis, and hip breadths, arm span, and right foot length were recorded (see Tab. S2b). Length of the upper limb (UL) and dimensions of the right hand were also measured (see Tab. S2c). Measures 1, 3, 4, 6, 7, 10, 11, 15, and 18 were gathered to establish individual

anthropometrically aligned actor (body) profiles within the MoCap acquisition software. Adhering to the recommendations from the MoCap technology manufacturer[31], this approach aims to ensure precision in MoCap data by optimizing the fit of the actor representation to the individual body dimensions of the participant. Previous study also showed that integration of measures 1, 3, 8, 9, and 13 facilitates the creation of anthropometrically aligned avatars with realistic body shapes[20]. These measures can be processed to generate person-matched avatars based on Skinned Multi-Person Linear (SMPL) models[34]. Specific measures (2, 5, and 11) were utilized in tasks `r01-06` to determine reaching heights at the eye, shoulder and at least knee levels that were tailored to each individual. Measures 12–15 of the UL were specifically taken to derive information on functional arm length to determine convenient reaching areas[32] for each participant. With the exception of arm span, pelvis, and hip width (measures 3, 7, and 8), all measures were exclusively obtained from the right side of the body. The measures were taken between typical anatomical reference points[29,30,32]. Weight (see Tab. S1, `mass`) and other weight-related characteristics (e. g., percent of body fat and water, see Tab. S5) were acquired in both sessions using a scale equipped with bioelectrical impedance analysis.

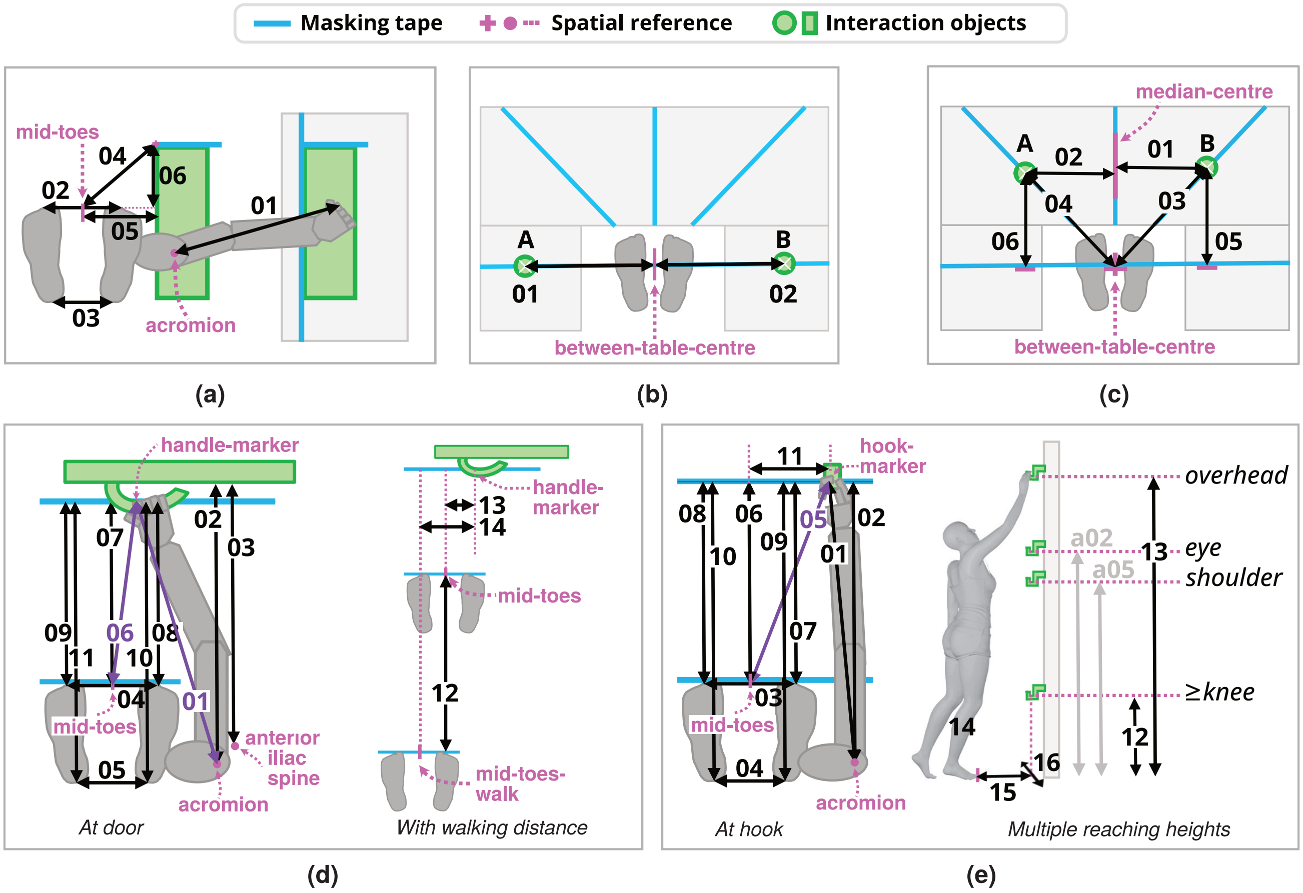

Figure S1. Overview of spatial measures based on individualized configuration of the spatial setup. (a) `e01-05`, (b) `g01`, (c) `h01`, (d) `o01-06`, and (e) `r01-08` (cf. Tab. 2). See Data Records for details.

Spatial setup configuration

To capture natural movement variations, the experimental setup for tasks involving object interactions was individually configured for each participant based on their anthropometric characteristics (see Tab. 2, `e01-05`, `g01`, `h01`, `o01-06`, `r01-08`). This individual customization involved participants adjusting the spatial setup to their natural upright posture and ease of reach, while also aligning it with their anthropometric measures such as shoulder, eye, and knee reaching heights (see Fig. S1e, cf. Tab. 2, `r01-08`). Specifically, to allow for individual adjustments, participants were introduced to the experimental setup and asked to fine-tune their standing positions and movement ranges within defined limits until they reached a configuration that suited their individual comfort levels. Spatial markers were placed to preserve these individual configurations for subsequent MoCap recording and were taken using a tape measure and rangefinder (Atolla; stated accuracy ±

2 mm) to ensure consistency across recording sessions. A detailed description can be found in the session protocols published with the dataset (see Data Records).

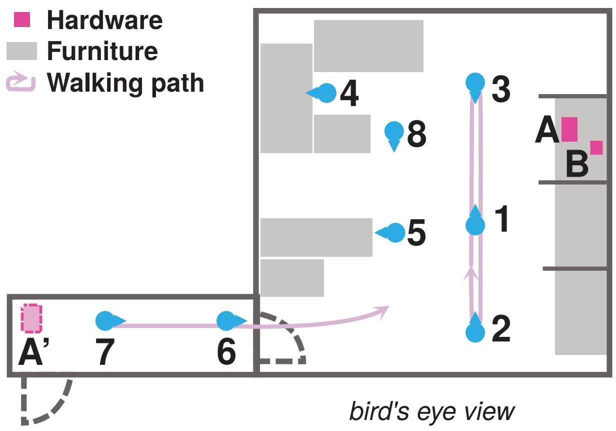

Task label Identifier		Recording spots
c01	→	1
e01–05	→	8
g01–02	→	4
h01–02	→	4
o01–02	→	6
o03–06	→	7–6–(1)
r01–08	→	5
s01	→	1
u01	→	1
w01–04	→	2–1–3, 3–1–2

Figure S2. Overview of the MoCap space. Spatial scheme of the task environment in MoCap space with recording spots 1–8 indicating the direction participants were facing during the motion execution of the associated tasks. Additional hardware consisted of a mobile laptop (A, A’) and a WLAN router (B).

Kinematic MoCap data

Due to the MoCap system’s sensitivity to electromagnetic interference, the MoCap space in our lab was designed to ensure a safe distance from electronic devices. The system was utilized within a space covering approximately 14.7 square meters (see Fig. S2), which included a main area (recording spots 1 to 5 and 8), as well as an interior hallway (recording spots 6 and 7). The MoCap data was captured using Rokoko Studio Legacy software (version 1.20.5r)[35] and was streamed from the data suit and gloves to a dedicated WLAN router (Asus RT-AC86U model). The acquisition laptop (HP Pavilion, model 15-ec2279ng) was primarily stationed in the main room next to the router, except for recording spots 6 and 7, where it was temporarily relocated into the hallway (see A’ in Fig. S2).

4 Additional information on data records

Overview of the dataset, including runs by task

This dataset includes 23 tasks with a single run each and seven tasks with multiple runs (totaling 26 recordings from `c01`: 2 runs; `o03-06`: 4 tasks × 5 runs each; `s01`: 2 runs; `u01`: 2 runs; cf. Tab. 2). On the example of TSV data, the dataset incorporates 32 participants × 49 recordings each, resulting in theoretically 1,568 TSV recordings in total. After subtracting 5 recordings removed during data pre-processing (see next section), the data set includes 1,563 TSV MoCap recordings, along with an equal number of BVH MoCap recordings.

Recordings removed due to specific issues

`sub-d02`, `r03` encountered connectivity problems that resulted in frame-wise data loss, `sub-d15`, `r08` showed an error during recording, causing the absence of the desired movement, and `sub-32`, `e03`, `e04`, and `e05` exhibited tracking inconsistencies in sensors of the upper body and hips.

The theory of Joint Coordinate Systems (JCSs) for describing rotational MoCap data

The International Society of Biomechanics (ISB) provides a widely recognized terminology for describing joint movements and coordinate systems, which enhances consistency and reproducibility of biomechanical measurements and terminology[36–39]. The calculation of rotational data is based on Joint Coordinate Systems (JCSs), which are customized for each joint to represent joint angles as relationships between the axes of the body segments connected by the joint. The ISB standard[40,41] and previous foundational works[42–44] explain the general principles for defining a JCS and provide specific guidelines for constructing a JCS for each joint, e.g., for the ankle, hip, or knee joint. JCSs are constructed in a standardized manner, with two body-fixed axes aligned with the anatomical landmarks of the proximal (denoted as axis "e_1"[42,43]) and distal (axis "e_3"[42,43]) body segments, and one floating axis (stated as "e_2"[42,43]) that is perpendicular to the two body-fixed axes. Zero values of e_1, e_2, and e_3 represent the neutral configuration of the joint [40, section 3.2.4]. This general convention in establishing the axes e_1 and e_3 with reference points on the distal and proximal body segments and defining e_2 as the floating axis ensures that JCSs align with medical terminology and provide anatomically meaningful and reproducible descriptions of joint motion[44].

To provide an applied example, the rotation of the ankle (talocrural) joint is defined as the motion of the foot (calcaneus) segment relative to the lower leg (tibia/fibula) segment[40]. Specifically, the Joint Coordinate System (JCS) for the ankle joint is defined as follows (see [40, section 3.5]): axis e_1 is fixed to the distal segment (the lower leg, identified as tibia/fibula), axis e_3 to the proximal segment (the foot, stated as calcaneus), and the floating axis e_2 is established perpendicular to e_1 and e_3. The rotation about e_1 represents *dorsiflexion* for positive values, and *plantarflexion* for negative values (see [40, section 3.5]). In the rotational MoCap data in the TSV files (see S-IDs 4 and 8 in Tab. S3), this motion is labelled as `LeftAnkle_dorsiflexion` and `RightAnkle_dorsiflexion` for the left and right ankle, respectively, with representing *dorsiflexion* for positive values, and *plantarflexion* for negative values (although the latter is not explicitly stated in the TSV joint label). Thus, the TSV joint labels in Tab. S3 represent bidirectional movements, even though only one direction is explicitly stated in the labels. Focusing on the example, the sensor data of the right and left ankle (see S-IDs 4 and 8 in Tab. S3) incorporates each 3 joint labels with each label describing movements in two opposite directions [40, section 3.5]: (i) `{Left|Right}Ankle_dorsiflexion` denoting dorsiflexion (values > 0) and plantarflexion (values < 0) for rotations about axis e_1, (ii) `RightAnkle_inversion` describing inversion (values > 0) and eversion (values < 0) for rotations about axis e_2, and (iii) `RightAnkle_internal_rotation` depicting internal (values > 0) as well as external rotation (values < 0) for rotations about axis e_3. Zero values of e_1, e_2, and e_3 represent the neutral configuration of the joint [40, section 3.2.4].

This method of defining JCSs by aligning them with the proximal and distal body segments provides an anatomically accurate and reproducible framework for reporting joint motion[44] that is consistently reflected for all joints in the TSV joint labels in Tab. S3, as specified in the ISB standard[40,41]. Note that for the knee joints (see S-IDs 3 and 7 in Tab. S3), flexion is represented by negative values and extension by positive values, which differs from the convention described by Grood and Suntay[42].

S-ID	Body location	TSV segment label	TSV joint label
1	L pelvis∗	Pelvis_position_{x|y|z}	Pelvis_{extension|lateral_flexion_rotation|axial_rotation}
2	L upper leg	LeftUpperLeg_position_{x|y|z}	LeftHip_{flexion|adduction|external_rotation}
3	L lower leg	LeftLowerLeg_position_{x|y|z}	LeftKnee_{flexion|adduction|external_rotation}
4	L foot	LeftFoot_position_{x|y|z}	LeftAnkle_{dorsiflexion|inversion|internal_rotation}
5	R pelvis∗	Pelvis_position_{x|y|z}	Pelvis_{extension|lateral_flexion_rotation|axial_rotation}
6	R upper leg	RightUpperLeg_position_{x|y|z}	RightHip_{flexion|adduction|external_rotation}
7	R lower leg	RightLowerLeg_position_{x|y|z}	RightKnee_{flexion|adduction|external_rotation}
8	R foot	RightFoot_position_{x|y|z}	RightAnkle_{dorsiflexion|inversion|internal_rotation}
9	L chest∗	Chest_position_{x|y|z}	Thorax_{extension|lateral_flexion_rotation|axial_rotation}
10	L shoulder	LeftShoulder_position_{x|y|z}	LeftScapula_{protraction|medial_rotation|posterior_tilt}
11	L upper arm	LeftUpperArm_position_{x|y|z}	LeftShoulder_{flexion|abduction|external_rotation}
12	L fore arm	LeftForeArm_position_{x|y|z}	LeftElbow_{flexion|abduction|pronation}
13	L hand	Substituted by S-ID 20	
14	R chest∗	Chest_position_{x|y|z}	Thorax_{extension|lateral_flexion_rotation|axial_rotation}
15	R shoulder	RightShoulder_position_{x|y|z}	RightScapula_{protraction|medial_rotation|posterior_tilt}
16	R upper arm	RightUpperArm_position_{x|y|z}	RightShoulder_{flexion|abduction|external_rotation}
17	R fore arm	RightForeArm_position_{x|y|z}	RightElbow_{flexion|abduction|pronation}
18	R hand	Substituted by S-ID 26	
19	head	Head_position_{x|y|z}	Neck_{flexion|left-ward_tilt|right-ward_rotation}
20	L wrist	LeftHand_position_{x|y|z}	LeftWrist_{flexion|abduction|pronation}
21–25	L fingers	n/a	n/a
26	R wrist	RightHand_position_{x|y|z}	RightWrist_{flexion|abduction|pronation}
27–31	R fingers	n/a	n/a
32	L hand	n/a	n/a
33	R hand	n/a	n/a

Table S3. Sensor identifier (S-ID) and location of the sensors (L denoting left, R denoting right body location) distributed in the textile of the data suit (S-IDs 1–19) and gloves (S-IDs 20–33), with corresponding labels for segments and joints as provided by the manufacturer and stored in the TSV files in accordance to the ISB standard[40–42]. Symbol ∗ marks interpolated data from multiple sensors. Finger data (S-IDs 21–25, 27–31) is not part of the TSV files, but is included in the BVH files (see Tab. S4).

5 BVH bone labels and hierarchy

S-ID	Body location	BVH bone label
1	L pelvis∗	Hips
2	L upper leg	LeftUpLeg
3	L lower leg	LeftLeg
4	L foot	LeftFoot
5	R pelvis∗	Hips
6	R upper leg	RightUpLeg
7	R lower leg	RightLeg
8	R foot	RightFoot
9	L chest∗	Spine, Spine1
10	L shoulder	LeftShoulder
11	L upper arm	LeftArm
12	L fore arm	LeftForeArm
13	L hand	Substituted by S-ID 20
14	R chest∗	Spine, Spine1, Spine2
15	R shoulder	RightShoulder
16	R upper arm	RightArm
17	R fore arm	RightForeArm
18	R hand	Substituted by S-ID 26
19	head	Neck
20	L wrist	LeftHand
21	L thumb	LeftHandThumb1, LeftHandThumb2
22	L index finger	LeftHandIndex1, RightHandIndex2
23	L middle finger	LeftHandMiddle1, RightHandMiddle2
24	L ring finger	LeftHandRing1, RightHandRing2
25	L pinky	LeftHandPinky1, RightHandPinky2
26	R wrist	RightHand
27	R thumb	RightHandThumb1, RightHandThumb2
28	R index finger	RightHandIndex1, RightHandIndex2
29	R middle finger	RightHandMiddle1, RightHandMiddle2
30	R ring finger	RightHandRing1, RightHandRing2
31	R pinky	RightHandPinky1, RightHandPinky2
32	L hand	NA
33	R hand	NA

BVH hierarchy (excerpt)

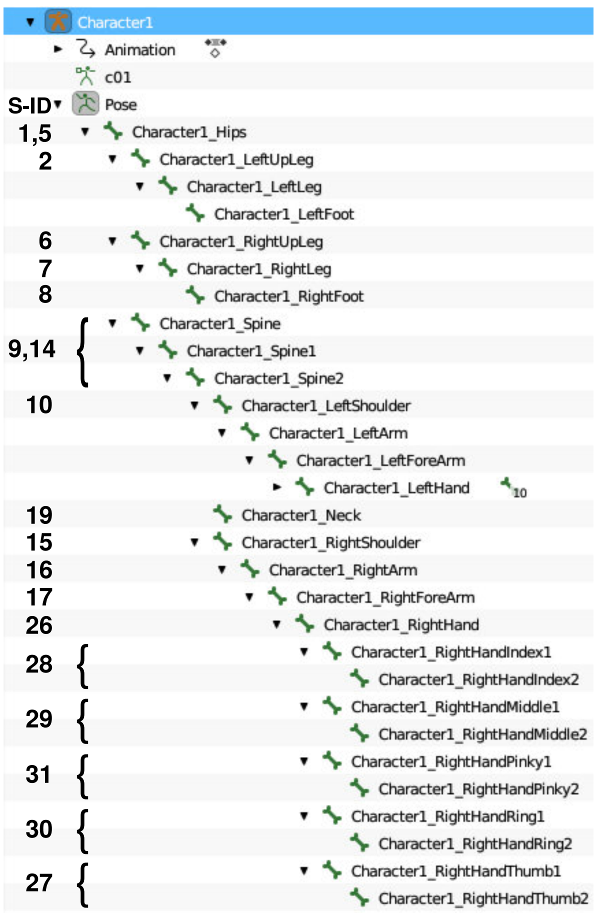

Table S4. BioVision Hierarchical motion capture data (BVH) bone labels and hierarchy, sorted by sensor identifier (S-ID) and sensor location – either for the right (R) or left (L) body side – for the data suit (S-IDs 1–19) and gloves (S-IDs 20–33). Symbol ∗ marks interpolated data from multiple sensors.

6 Column field names in the `participants.tsv` file

The `participants.tsv` file contains demographic, anthropometric, and other data, as listed in Tab. S5, and stated next:

- *Basic demographic data* include the `participant_id`, which is reflected in the participant subdirectory naming template (e. g., *sub-d05/*); `participant_group` (`OA` for older adults or `YA` for younger adults); sex (`m` for male or `f` for female); age in years; and handedness (`right` or `left`).
- *Extended demographic data* include the EHI laterality quotient of handedness `ehi_lq`[45]; the execution times and other characteristics of the 5RSTST (task `s01` in Tab. 2) for both runs (e.g, `sts_01_time` and `sts_02_time`, respectively); and self-reported medical conditions that might affect the participants' ability to execute movements effectively (see Tab. S5).
- *Anthropometric body measures* are described by `a{01-18}`, with `a` as the prefix and a zero-padded number from `01` to `18` according to Tab. S2. For example, `a01` refers to body height (stature). Weight-related measures are averaged over both sessions, stored with the prefix `mass` (cf. Tab. S1).
- *Individualized spatial configurations* record individual adjustments of the experimental setup for tasks involving object interactions, represented by `{E|G|H|O|R}_<number>(_dir)`. The uppercase corresponds to the lowercase letter in the TL-ID in Tab. 2, and the `<number>` specifies the spatial measures available for particular tasks, as detailed in Fig. S1. For example, `R_13` indicates the height of the overhead reaching target for tasks `r07-08` (see Fig. S1e). Individualized configurations were recorded for the following tasks (see Fig. S1 and Tab. S5): `E_{01...06}` for tasks `e01-06`; `G_{01...02}` for task `g01`; `H_{01...06}` for task `h01`; `O_{01...11}` for tasks `o01-02`, `O_{12...14}`, `O_13_dir`, and `O_14_dir` for tasks `o03-06`. Reaching tasks utilize `R_{1...11}` and `R_11_dir` for the initial standing position in tasks `r01-08` and `R_{12...16}` for the different reaching heights in tasks `r04-08`.
- *Motion tracking quality* The experimenter assessed the quality of motion tracking of the data suit and gloves trough visual observation, comparing the participants' movements to those of the live avatar in the MoCap acquisition software. Drift in the avatar's global position, inaccurate positions or rotations of the avatar's body segments, and missing or faulty sensor signals were documented. Motion tracking quality was denoted by `tracking_{TL-ID}_{run_index_or_range}`. For example, the column `tracking_c01_01` records the tracking quality in the calibration task `c01` in run 1, while `tracking_o03_01-05` states the tracking quality in the door interaction task `o03` across runs 1–5 (see Tab. 2). Quality was assessed by the experimenter as `ok` (no issues), `missing` (removed recordings), or as a list of affected body regions, i. e., `head-neck`, `torso-spine`, `arms-shoulders`, `hands-wrists`, `hips-legs`, or `ankles-feet` (multiple entries possible). The column `tracking_comment` contains free-form notes from the experimenter, explaining tracking issues or missing data (affects sub-d02, `r03`; sub-d15, `r08`; and sub-d32, `e03-05`).
- *Additional demographic data* include participant information on *educational background*, *history of pain* acquired by using the NMQ[46,47], *physical activity*, and *garment sizes*. Please refer to Tab. S5 and the file `participants.json`.

Column range	Field names
[0:5)	**Basic demographic data** participant_id, participant_group, sex, age, handedness
[5:28)	**Extended demographic data** ehi_lq, sts_01_time, sts_02_time, has_passed_sts_01, correct_style_sts_01, has_passed_sts_02, correct_style_sts_02, eye_sight, hearing, is_treated_with_kinesiotape, has_prothesis_orthosis, has_foot_deformity, has_foot_orthotics, has_dizziness, has_osteoporosis, has_gout, has_rheumatism, has_multiple_sclerosis, has_parkinson, has_poliomyelitis, has_scoliosis, has_stroke, other_medical_limitations
[28:31)	**Educational background** level_education, is_employed, employment_type
[31:48)	**Physical activity** sitting_activity, is_active_sports, activity_per_week, moderate_activity, intense_activity, does_sports_cycle, does_sports_dance, does_sports_football, does_sports_golf, does_sports_gymyoga, does_sports_handball, does_sports_jog, does_sports_martial_arts, does_sports_ride, does_sports_swim, does_sports_tennis, does_sports_misc
[48:58)	**Sizes of data suit and gloves and other garment sizes** suit_size_s01, suit_size_s02, gloves_size_s01, gloves_size_s02, size_top, size_bottom, size_shoes, size_jeanslength, size_jeanswidth, size_type
[58:82)	**Anthropometric and weight-related data** a01, a02, a03, a04, a05, a06, a07, a08, a09, a10, a11, a12, a13, a14, a15, a16, a17, a18, mass, mass_body_fat, mass_water, mass_muscle, mass_fat_index, bmi
[82:118)	**History of pain** pain_01_neck_last_year, pain_02_upperspine_last_year, pain_03_lowerspine_last_year, pain_04_shoulders_last_year, pain_05_elbows_last_year, pain_06_hands_last_year, pain_07_hips_last_year, pain_08_knees_last_year, pain_09_feet_last_year, has_pain_01_neck_affected_life, has_pain_02_upperspine_affected_life, has_pain_03_lowerspine_affected_life, has_pain_04_shoulders_affected_life, has_pain_05_elbows_affected_life, has_pain_06_hands_affected_life, has_pain_07_hips_affected_life, has_pain_08_knees_affected_life, has_pain_09_feet_affected_life, pain_01_neck_last_week, pain_02_upperspine_last_week, pain_03_lowerspine_last_week, pain_04_shoulders_last_week, pain_05_elbows_last_week, pain_06_hands_last_week, pain_07_hips_last_week, pain_08_knees_last_week, pain_09_feet_last_week, pain_01_neck_since_s01, pain_02_upperspine_since_s01, pain_03_lowerspine_since_s01, pain_04_shoulders_since_s01, pain_05_elbows_since_s01, pain_06_hands_since_s01, pain_07_hips_since_s01, pain_08_knees_since_s01, pain_09_feet_since_s01
[118:167)	**Spatial setup configurations** E_01, E_02, E_03, E_04, E_05, E_06, E_06_dir, G_01, G_02, H_01, H_02, H_03, H_04, H_05, H_06, O_01, O_02, O_03, O_04, O_05, O_06, O_07, O_08, O_09, O_10, O_11, O_12, O_13, O_13_dir, O_14, O_14_dir, R_01, R_02, R_03, R_04, R_05, R_06, R_07, R_08, R_09, R_10, R_11, R_11_dir, R_12, R_13, R_14, R_15, R_16, R_16_dir
[167:201)	**Motion tracking quality** tracking_c01_01, tracking_c01_02, tracking_e01_01, tracking_e02_01, tracking_e03_01, tracking_e04_01, tracking_e05_01, tracking_g01_01, tracking_g02_01, tracking_h01_01, tracking_h02_01, tracking_o01_01, tracking_o02_01, tracking_o03_01-05, tracking_o04_01-05, tracking_o05_01-05, tracking_o06_01-05, tracking_r01_01, tracking_r02_01, tracking_r03_01, tracking_r04_01, tracking_r05_01, tracking_r06_01, tracking_r07_01, tracking_r08_01, tracking_s01_01, tracking_s01_02, tracking_u01_01, tracking_u01_02, tracking_w01_01, tracking_w02_01, tracking_w03_01, tracking_w04_01, tracking_comment

Table S5. Overview of column field names in the `Participant.tsv` file. The column ranges can be used for data slicing, with intervals represented as $[a:b)$, where the range starts inclusively at number a and continues up to, but not including, number b.

7 Additional validation approaches

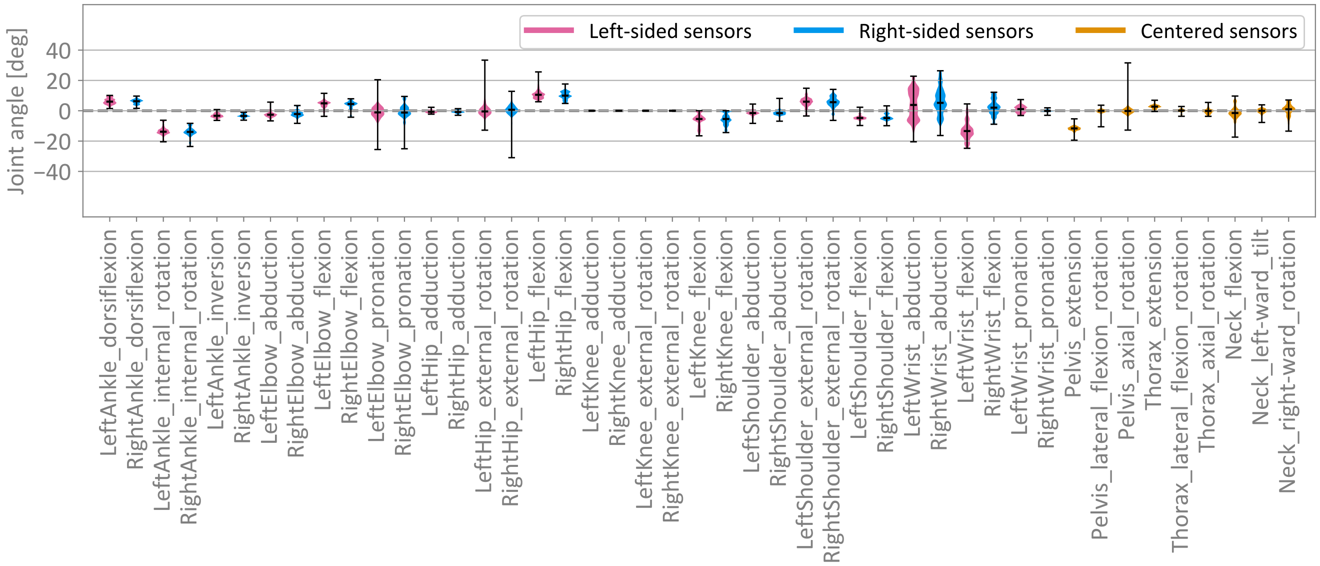

Figure S3. Joint angle distribution for the calibration pose for task `c01` in run 01 across all participants. The dashed gray line indicate the zero-degree reference; and the standard error bars associated with each sensor indicates variability across participants.

Joint angle distribution during calibration

We checked and validated the calibration pose (task `c01`; see Tab. 2, no. 1) because its crucial role in calibrating the measurements. The calibrated rotation values, in terms of joint angles, should ideally be close to zero for most sensors. Fig. S3 depicts the violin plots for all sensors of the data suit (excluding scapula joints S-IDs 11 & 16) across all participants for a single run (run index 01) of the calibration pose. We focused our analysis on the middle section of the raw MoCap recording, specifically on frames corresponding to the 45th to 55th percentiles of the total frames, during which the calibration pose was executed. This selection aimed to mitigate transient effects, such as initial posture adjustments and potential bias that might arise at the beginning or end of the recording.

The results shown in Fig. S3 indicate that the majority of sensors display zero-centred (mean) values. We excluded the left and right scapula sensors from this analysis because, according to the ISB, the scapular coordinate system is defined in alignment with the clavicular coordinate system and does not accurately reflect the true anatomical position of the scapula [41, p. 985]. A few sensors exhibit larger deviations from the zero-degree reference (horizontal dashed-gray line in Fig. S3) in certain anatomical directions, but these all remain within tolerable range of $\pm$ 20 degrees based on previous studies, covering the deviations of the pelvis and hip joints[48,49], ankles[50], and knees[51], as explicated next. Notable deviations occur in the extension/flexion of the hips, pelvis, left wrist, and both knees, as well as in the internal/external rotation of the shoulders and ankles. They may result from a natural upright posture that differs from an ideally executed calibration pose. In a typical upright stance, the pelvis is usually inclined forward by approximately 12 degrees[48]. This condition, known as anterior pelvic tilt, is very common and exhibits significant variability across studies[49]. Anterior pelvic tilt is evident in `Pelvis_extension` in Fig. S3 (reflecting pelvic flexion/forward tilt by -11.5 $\pm$ 1.9 degrees), and in the left and right hip (see `{Left|Right}Hip_flexion` in Fig. S3).

Previous research on preferred foot placement during quiet stance has shown that the average stance angle (the angle between the long axes of the feet from the center of the heels to the first toes) is 14 degrees across all ages[50]. Although participants were trained on how to execute the calibration pose correctly, they may have naturally adopted a comfortable foot position, reflected in a mean external rotation of 13.8 $\pm$ 2.8 degrees for the left ankle and 13.9 $\pm$ 3.3 degrees for the right ankle (see `Left|RightAnkle_internal_rotation` in Fig. S3, negative values reflect external rotation, see Data Records). Such feet orientation aligns with typical behavior during (comfortable) upright stance. Additionally, individuals may naturally exhibit slight knee flexion (see `{Left|Right}Knee_flexion` in Fig. S3, for the left knee -5.7 $\pm$ 4.5, and for the right -5.1 $\pm$ 4.0), which enhances postural stability[51]. Variability in the external rotation and flexion of the shoulders may be attributed to natural differences in arm positioning or habitual shoulder posture.

Although perfect posture symmetry is very rarely observed[52], the here observed symmetrical deviations from the zero reference on both the left and right side of the body indicate low inter-limb kinematic differences as found in past research on

natural movement behavior[53]. On the contrary, the deviation observed only in the left wrist is particularly noteworthy (see `Left|RightWrist_flexion` in Fig. S3, where the left wrist shows -12.9 $\pm$ 6.3 degrees of extension in comparison to a flexion of 2.2 $\pm$ 5.1 degrees in the right wrist). This asymmetry is unlikely due to measurement error, as gloves were tailored to participants' dimensions (see Anthropometry), and only bimanual hand use (see Ses-ID 01 in Tab. 2) was recorded, excluding fatigue as a factor. The left wrist's extension could be related to handedness, possibly due to exaggeration during calibration, while the right wrist remained slightly flexed.

A few sensors showed greater variations across participants (indicated by the larger standard error bars around the means in Fig. S3), notably in the arms (elbow pronation/supination, wrist flexion/extension, and abduction/adduction), torso (external rotation of hips and pelvis axial rotation), and neck (flexion/extension and lateral left/right-ward rotation). The variability during static calibration of IMU sensors is a well-recognized issue[54]. Similar variations in elbow angles across participants have been observed in previous research[55]. Variability in wrist and neck orientation, along with elbow pronation, could be attributed to the considerable mobility of the hands and neck, as well as minor differences in hand and head positions during calibration. Since the position of the vestibular organ within the head does not significantly influence postural control[56], participants may have adopted different head orientations. Additionally, in older participants, postural misalignments, such as those affecting the cervical spine and head posture, could further impact the execution of the calibration pose[57,58].

8 Segmentation

Overview of the processing segmentation pipeline

The processing pipeline subdivides movements (on the example of reaching tasks) into meaningful segments by applying the following steps (see Fig. S4, shown for task `r01`): (i) detecting peak values in the data suitable for segmenting it (see Fig. S4b); (ii) refining and filtering the peak values and utilizing them to segment the data (see Fig. S4c), specifically for creating segments that encompass forward motion phases with object transfer (cf. Fig. 3c) or backward motion phases without object transfer; (iii) translating the segments to approx. alignment with the coordinate origin to reduce positional variability in motion execution (see Fig. S4d); (iv) normalizing the segments by the mean (vector) magnitude across all segments to ensure a common value range (see normalized space in the range $[0,1]$ in Fig. S4e); and (v) resampling the segments to achieve a stable number of sampled points per segment (not depicted in Fig. S4). For visual inspection and interpretation, the motion trajectories can be plotted after undergoing the aforementioned processing steps (see Fig. S4f). When comparing the processed and segmented data to the position coordinates before applying the processing steps (see Fig. S4a and Fig. S4f), several noticeable differences can be observed. The processed data exhibits comparable value ranges, with starting points centered around the coordinate origin, resulting in trajectories that begin from a similar reference point. Additionally, the trajectory lengths are also comparable across participants. These visualizations can serve as a starting point for a more detailed interpretation and validation of the motion trajectories. In the subsequent sections, we interpret the graphs in more details.

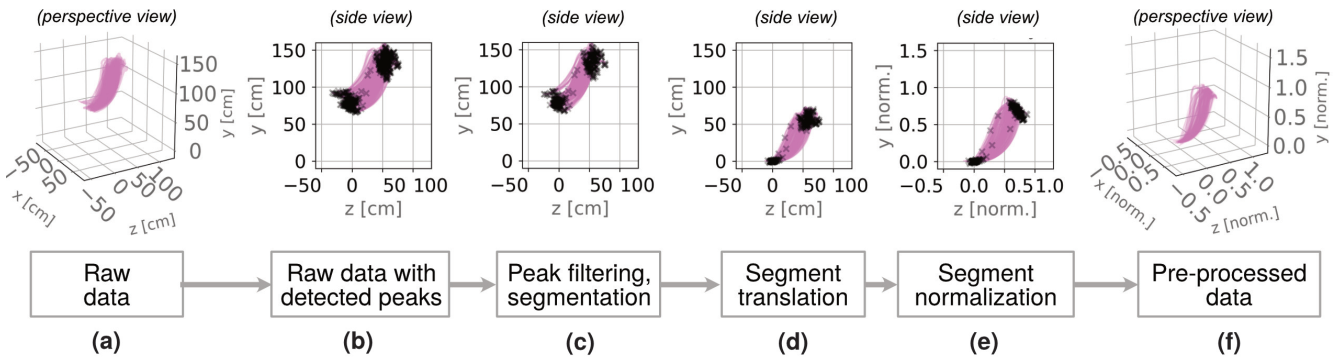

Figure S4. Processing pipeline on a selected example of task `r01` (placing a towel on a hook in shoulder height and retrieving it). The position of the `RightHand` (in pink) over all participants is displayed in either ((a), (f)) 3-dimensional perspective, involving the (x,y,z) values, or ((b)–(e)) side view, displaying only the (z,y) values of the sensor. To prepare the data for further analysis and interpretation, (b) peak values in the signal are detected (shown as cross symbols), (c) refined and filtered, and used to crop the signal into segments. The segments are (d) translated, (e) normalized, and resampled (not displayed). (f) At the end of the processing pipeline, the spatial representation of reaching trajectories over participants is aligned and the influence of spatial individual characteristics is reduced.

Validation of segmented movements in a task

This pipeline processes only the positional data, i. e., the (x,y,z) values (see Data Records). While the processing steps were tailored to these reaching tasks `r01-03`, and `r07`, they can be adapted for other tasks with different characteristics. Fig. S4a shows the 3d motion trajectories of the `RightHand_position` across all participants in task `r01`. The unprocessed position values show variability from different standing positions of the participants, individual arm lengths, and individualized target positions while performing the upward-forward reach. For clarity, segmentation steps are shown for (z,y) values (see Fig. S4b, cf. Fig. 7), with a focus on the right wrist sensor data (see S-ID 26 in Tab. S3).

- **Detecting peaks.** First, peak values in the `position_z` component (reflecting movements towards or away from the target, see Data Records) of the right wrist were detected. The peak values in the data were obtained separately for different points in time during the motion execution, reflecting the key poses when standing in neutral pose and when reaching the target. The first set of peaks, termed $peaks^{neutral}$ in Fig. 5a represent the starting points of repetitions and phases with forward motion (cf. Fig. 3). The second set of peaks, termed $peaks^{target}$ in Fig. 5a (cf. Fig. 3), corresponds to the points in time when the target is reached. Fig. S4b depicts the result of the peak detection step, displaying two raw clusters of peaks indicated by black markers. The lower left cluster indicates the peaks at starting position, while the upper right cluster represents the peaks when the target was reached. Faint lines in pink represent the movement trajectories.
- **Filtering and refining peaks.** Since various factors, such as object characteristics, can influence the motion path (see

Section 2, Design considerations of selected motion tasks), additional filtering was applied to detected peaks. In this case, only the peaks that occur in phases with forward motion while carrying an object are selected for further analysis (see t_1, t_2 in Fig. 3e). Before filtering, a peak refinement method was applied only for these motion performances where no starting peak for the first segment was detected. This could be the case for recordings of participants who started rapidly without holding the neutral pose for some milliseconds. Subsequently, motion segments were created from the filtered peaks (see segments $s_1, s_5, s_9, s_{13}, s_{17}$ in Fig. 5c to Fig. 5e), resulting in 5 segments of forward motion with object transfer per participant (for each processed reaching task). Fig. S4c displays the result of this filtering and segmenting procedure, resulting in a reduced number of trajectories compared to Fig. S4b.

During data collection the spatial setup of the tasks `r01-08` was customized to the individual participants. The variations in starting and target positions between participants (illustrated in Fig. S4c), are a result of differences in arm length and shoulder levels. It is important to also consider participant-specific adjustments and movement variability that may occur over repetitions due to spatial and temporal adaptations in motion execution. These adjustments are visually observable in Fig. 8 (panel A) where postural differences are evident in the BVH skeletal representation. Additionally, this variability is reflected in Fig. 8 (panel B) which displays changes in the starting and ending $(z-y)$ positions of the reaching trajectories (as discussed previously in that section). To address these positional variations, the following processing steps take into account inter- and intra-individual differences and thus ensure comparability in subsequent analysis.

- **Translation of starting positions.** The positional values in the motion trajectories are normalized to a common range by centering their starting positions around the origin for all participants. To achieve this, the mean (x, y, z) position of the right wrist (see sensor labelled with S-ID 26 in Tab. S3) was calculated for each participant at the time frames corresponding to the $peaks^{neutral}$. This mean value was then subtracted from all sensor positions of all segments, retaining slight variance in the starting positions characteristic for a natural motion execution. It is worth noting that while the positioning of the right wrist in the neutral pose should be similar across segments, it is not expected to be identical. Therefore, the normalized position values of the wrist sensor at the beginning of a segment should be centered around the origin (see black markers in Fig. S4d). The normalization approach used here for the right wrist sensor as an illustration can also be applied to other sensors in the dataset.
- **Normalization of trajectory length.** In the illustrative example of the right wrist sensor, further standardization was applied to the normalized data by scaling the trajectory length to account for the individual reaching distances of the participants. This was achieved by calculating the mean vector length (magnitude) of the reaching trajectories for each participant. By determining the average length of the vectors, an individualized normalization factor was obtained. For each participant, the position values of each segment within the trajectory were then divided by this mean length. Consequently, the resulting normalized vector lengths are smaller than 1, reflecting the relative proportion of the participant's reaching distance with respect to their mean length. Fig. S4e illustrates the normalized reaching trajectories across all participants, with comparable (but not numerically equal) starting and ending positions of the trajectories.
- **Resampling.** In a final processing step, the data underwent resampling to ensure an equal number of sample points across all segments and participants. This uniformity facilitates the application of ML algorithms, which typically require consistent input dimensions.

References

1. Choi, Y. *et al.* Translating AI to Clinical Practice: Overcoming Data Shift with Explainability. *RadioGraphics* **43**, e220105, https://doi.org/10.1148/rg.220105 (2023).
2. Chen, K. *et al.* Deep Learning for Sensor-based Human Activity Recognition: Overview, Challenges and Opportunities, https://doi.org/10.48550/arXiv.2001.07416 (2021). 2001.07416.
3. Medeiros, H. B. D. O., Araújo, D. S. M. S. D. & Araújo, C. G. S. D. Age-related mobility loss is joint-specific: An analysis from 6,000 Flexitest results. *AGE* **35**, 2399–2407, https://doi.org/10.1007/s11357-013-9525-z (2013).
4. Fothergill, S., Mentis, H., Kohli, P. & Nowozin, S. Instructing people for training gestural interactive systems. In *Proceedings of the SIGCHI Conference on Human Factors in Computing Systems*, 1737–1746, https://doi.org/10.1145/2207676.2208303 (ACM, Austin Texas USA, 2012).
5. Hardwick, R. M. & Edwards, M. G. Observed reach trajectory influences executed reach kinematics in prehension. *Q. J. Exp. Psychol.* **64**, 1082–1093, https://doi.org/10.1080/17470218.2010.538068 (2011).
6. Chang, E., Caplinger, J., Marin, A., Shen, X. & Demberg, V. DART: A Lightweight Quality-Suggestive Data-to-Text Annotation Tool. In *Proc. 28th Int. Computat. Linguistics: System Demonstrations*, 12–17, https://doi.org/10.18653/v1/2020.coling-demos.3 (Int. Committee on Computat. Linguistics (ICCL), Barcelona, Spain (Online), 2020).
7. Song, Y. C. *et al.* Unsupervised Alignment of Actions in Video with Text Descriptions. In *Proc. 25th Int. Joint Conf. on Artif. Intellig. (IJCAI-16)*, 2025–2031 (2016).
8. Zhou, L., Li, W., Ogunbona, P. & Zhang, Z. Semantic action recognition by learning a pose lexicon. *Pattern Recognit.* **72**, 548–562, https://doi.org/10.1016/j.patcog.2017.06.035 (2017).
9. Punnakkal, A. R., Chandrasekaran, A., Athanasiou, N., Quiros-Ramirez, A. & Black, M. J. BABEL: Bodies, Action and Behavior with English Labels. *arXiv:2106.09696 [cs]*, https://doi.org/10.48550/arXiv.2106.09696 (2021).
10. Murphy, M. A., Sunnerhagen, K. S., Johnels, B. & Willén, C. Three-dimensional kinematic motion analysis of a daily activity drinking from a glass: A pilot study. *J NeuroEngineering Rehabil* **3**, 18, https://doi.org/10.1186/1743-0003-3-18 (2006).
11. Naghibi, S. S., Fallah, A., Maleki, A. & Ghassemi, F. Elbow angle generation during activities of daily living using a submovement prediction model. *Biol Cybern* **114**, 389–402, https://doi.org/10.1007/s00422-020-00834-w (2020).
12. Iachini, T. *et al.* The experience of virtual reality: are individual differences in mental imagery associated with sense of presence? *Cogn. processing* **20**, 291–298, https://doi.org/10.1007/s10339-018-0897-y (2019).
13. Chen, H.-c., Lin, K.-c., Chen, C.-l. & Wu, C.-y. The Beneficial Effects of a Functional Task Target on Reaching and Postural Balance in Patients with Right Cerebral Vascular Accidents. *Mot. Control.* **12**, 122–135, https://doi.org/10.1123/mcj.12.2.122 (2008).
14. Wisneski, K. J. & Johnson, M. J. Quantifying kinematics of purposeful movements to real, imagined, or absent functional objects: Implications for modelling trajectories for robot-assisted ADL tasks**. *J NeuroEngineering Rehabil* **4**, 7, https://doi.org/10.1186/1743-0003-4-7 (2007).
15. Duncan, P. W., Weiner, D. K., Chandler, J. & Studenski, S. Functional Reach: A New Clinical Measure of Balance. *J. Gerontol.* **45**, M192–M197, https://doi.org/10.1093/geronj/45.6.M192 (1990).
16. Row, B. S. & Cavanagh, P. R. Reaching upward is more challenging to dynamic balance than reaching forward. *Clin. Biomech.* **22**, 155–164, https://doi.org/10.1016/j.clinbiomech.2006.06.003 (2007).
17. Eriksrud, O., Federolf, P. A. & Cabri, J. Influence of Anthropometry, Age, Sex, and Activity Level on the Hand Reach Star Excursion Balance Test. *Front. Psychol.* **10**, 756, https://doi.org/10.3389/fpsyg.2019.00756 (2019).
18. Johnston, H., Dewis, C. & Kozey, J. Comparison Considerations Toward Investigating the Factors of Load and Age Group on the Maximum Reach Envelope. *Hum Factors* **64**, 785–799, https://doi.org/10.1177/0018720820965018 (2022).
19. Samson, M. M. *et al.* Differences in gait parameters at a preferred walking speed in healthy subjects due to age, height and body weight. *Aging Clin Exp Res* **13**, 16–21, https://doi.org/10.1007/BF03351489 (2001).
20. Pujades, S. *et al.* The Virtual Caliper: Rapid Creation of Metrically Accurate Avatars from 3D Measurements. *IEEE Trans. Vis. Comput. Graph.* **25**, 1887–1897, https://doi.org/10.1109/TVCG.2019.2898748 (2019).
21. Reipschläger, P. *et al.* AvatAR: An Immersive Analysis Environment for Human Motion Data Combining Interactive 3D Avatars and Trajectories. In *CHI Conference on Human Factors in Computing Systems*, 1–15, https://doi.org/10.1145/3491102.3517676 (ACM, New Orleans LA USA, 2022).

22. Jung, M., Sim, S., Kim, J. & Kim, K. Impact of Personalized Avatars and Motion Synchrony on Embodiment and Users' Subjective Experience: Empirical Study. *JMIR Serious Games* **10**, e40119, https://doi.org/10.2196/40119 (2022).

23. Stergiou, M., El Raheb, K. & Ioannidis, Y. Imagery and metaphors: From movement practices to digital and immersive environments. In *Proceedings of the 6th International Conference on Movement and Computing*, 1–8, https://doi.org/10.1145/3347122.3347141 (ACM, Tempe AZ USA, 2019).

24. Kirby, R., Price, N. & MacLeod, D. The influence of foot position on standing balance. *J. Biomech.* **20**, 423–427, https://doi.org/10.1016/0021-9290(87)90049-2 (1987).

25. Promsri, A., Haid, T. & Federolf, P. Complexity, Composition, and Control of Bipedal Balancing Movements as the Postural Control System Adapts to Unstable Support Surfaces or Altered Feet Positions. *Neuroscience* **430**, 113–124, https://doi.org/10.1016/j.neuroscience.2020.01.031 (2020).

26. Srinivasan, D. & Martin, B. Object and target size interactions in placement tasks. *Proc. Hum. Factors Ergonomics Soc. Annu. Meet.* **52**, 940–944, https://doi.org/10.1177/154193120805201309 (2008).

27. Linkenauger, S. A., Witt, J. K., Stefanucci, J. K., Bakdash, J. Z. & Proffitt, D. R. The effects of handedness and reachability on perceived distance. *J. Exp. Psychol. Hum. Percept. Perform.* **35**, 1649–1660, https://doi.org/10.1037/a0016875 (2009).

28. Rosenbaum, D. A. Reaching while walking: Reaching distance costs more than walking distance. *Psychon. Bull. & Rev.* **15**, 1100–1104, https://doi.org/10.3758/PBR.15.6.1100 (2008).

29. ISO/TC 159 Ergonomics. ISO 7250-1 Basic human body measurements for technological design - Part 1: Body measurement definitions and landmarks. Tech. Rep. ISO 7250-1:2017(E), International Organization for Standardization, Geneva, Switzerland (2017).

30. Paquette, S., Gordon, C. & Bradtmiller, B. Anthropometric Survey (ANSUR) II Pilot Study: Methods and Summary Statistics. Tech. Rep. NATICK/TR-09/014, Anthrotech, Yellow Springs, OH 45387 (2009).

31. Rokoko Electronics. Actor Profile. https://support.rokoko.com/hc/en-us/articles/4410415403025-Actor-Profile.

32. Pheasant, S. *Bodyspace: Anthropometry, Ergonomics and the Design of Work* (CRC Press, 2003), 2 edn.

33. Rokoko Electronics. Which Smartgloves size is right for me? https://support.rokoko.com/hc/en-us/articles/14960461266961-Which-Smartgloves-size-is-right-for-me-.

34. Loper, M., Mahmood, N., Romero, J., Pons-Moll, G. & Black, M. J. SMPL: A skinned multi-person linear model. *ACM Trans. Graph.* **34**, 1–16, https://doi.org/10.1145/2816795.2818013 (2015).

35. Rokoko Electronics. Download rokoko studio. https://www.rokoko.com/products/studio/download.

36. Żuk, M. & Pezowicz, C. Kinematic Analysis of a Six-Degrees-of-Freedom Model Based on ISB Recommendation: A Repeatability Analysis and Comparison with Conventional Gait Model. *Appl. Bionics Biomech.* **2015**, 1–9, https://doi.org/10.1155/2015/503713 (2015).

37. Żuk, M. & Trzeciak, M. Anatomical protocol for gait analysis: Joint kinematics measurement and its repeatability. *jtam* 369, https://doi.org/10.15632/jtam-pl.55.1.369 (2016).

38. Gates, D. H., Walters, L. S., Cowley, J., Wilken, J. M. & Resnik, L. Range of Motion Requirements for Upper-Limb Activities of Daily Living. *Am. J. Occup. Ther.* **70**, 7001350010p1–7001350010p10, https://doi.org/10.5014/ajot.2016.015487 (2016).

39. Robert-Lachaine, X., Mecheri, H., Larue, C. & Plamondon, A. Accuracy and repeatability of single-pose calibration of inertial measurement units for whole-body motion analysis. *Gait & Posture* **54**, 80–86, https://doi.org/10.1016/j.gaitpost.2017.02.029 (2017).

40. Wu, G. *et al.* ISB recommendation on definitions of joint coordinate system of various joints for the reporting of human joint motion—part I: Ankle, hip, and spine. *J. Biomech.* **35**, 543–548, https://doi.org/10.1016/S0021-9290(01)00222-6 (2002).

41. Wu, G. *et al.* ISB recommendation on definitions of joint coordinate systems of various joints for the reporting of human joint motion—Part II: Shoulder, elbow, wrist and hand. *J. Biomech.* **38**, 981–992, https://doi.org/10.1016/j.jbiomech.2004.05.042 (2005).

42. Grood, E. S. & Suntay, W. J. A Joint Coordinate System for the Clinical Description of Three-Dimensional Motions: Application to the Knee. *J. Biomech. Eng.* **105**, 136–144, https://doi.org/10.1115/1.3138397 (1983).

43. Cole, G. K., Nigg, B. M., Ronsky, J. L. & Yeadon, M. R. Application of the Joint Coordinate System to Three-Dimensional Joint Attitude and Movement Representation: A Standardization Proposal. *J. Biomech. Eng.* **115**, 344–349, https://doi.org/10.1115/1.2895496 (1993).

44. Wu, G. & Cavanagh, P. ISB Recommendations for Standardization in the Reporting of Kinematic Data. *J. Biomech.* **28**, 1257–1261 (1995).

45. Oldfield, R. The assessment and analysis of handedness: The Edinburgh inventory. *Neuropsychologia* **9**, 97–113, https://doi.org/10.1016/0028-3932(71)90067-4 (1971).

46. Kuorinka, I. *et al.* Standardised Nordic questionnaires for the analysis of musculoskeletal symptoms. *Appl. Ergonomics* **18**, 233–237 (1987).

47. Caffier, G., Steinberg, U. & Liebers, F. Praxisorientiertes Methodeninventar zur Belastungs- und Beanspruchungsbeurteilung im Zusammenhang mit arbeitsbedingten Muskel-Skelett-Erkrankungen. Tech. Rep. Fb850, Bundesanstalt für Arbeitsschutz und Arbeitsmedizin, Dortmund/Berlin (1999).

48. Ryf, Chr. & Weymann, A. The neutral zero method — A principle of measuring joint function. *Injury* **26**, 1–11, https://doi.org/10.1016/0020-1383(95)90116-7 (1995).

49. Suits, W. H. Clinical Measures of Pelvic Tilt in Physical Therapy. *Int. J. Sports Phys. Ther.* **16**, https://doi.org/10.26603/001c.27978 (2021).

50. McIlroy, W. & Maki, B. Preferred placement of the feet during quiet stance: Development of a standardized foot placement for balance testing. *Clin. Biomech.* **12**, 66–70, https://doi.org/10.1016/S0268-0033(96)00040-X (1997).

51. Pereira, H. M. *et al.* Influence of knee position on the postural stability index registered by the Biodex Stability System. *Gait & Posture* **28**, 668–672, https://doi.org/10.1016/j.gaitpost.2008.05.003 (2008).

52. Masse, M., Gaillardetz, C., Cron, C. & Abribat, T. A new symmetry-based scoring method for posture assessment: Evaluation of the effect of insoles with mineral derivatives. *J. Manip. Physiol. Ther.* **23**, 596–600, https://doi.org/10.1067/mmt.2000.110946 (2000).

53. Hill, C. N., Ross, S., Peebles, A. & Queen, R. M. Continuous similarity analysis in patient populations. *J. Biomech.* **131**, 110916, https://doi.org/10.1016/j.jbiomech.2021.110916 (2022).

54. Donaldson, B., Bezodis, N. & Bayne, H. Within-subject repeatability and between-subject variability in posture during calibration of an inertial measurement unit system. In *ISBS Proc. Arch.*, vol. 39 of *1* (Canberra, Australia, 2021).

55. Morton, L., Baillie, L. & Ramirez-Iniguez, R. Pose calibrations for inertial sensors in rehabilitation applications. In *2013 IEEE 9th Int. Conf. Wirel. Mob. Comput. Netw. Commun. WiMob*, 204–211, https://doi.org/10.1109/WiMOB.2013.6673362 (IEEE, Lyon, France, 2013).

56. Hansson, E. E., Beckman, A. & Håkansson, A. Effect of vision, proprioception, and the position of the vestibular organ on postural sway. *Acta Oto-Laryngologica* **130**, 1358–1363, https://doi.org/10.3109/00016489.2010.498024 (2010).

57. Irani, S. *et al.* The Effect of Head and Neck Stabilization Exercises on Dynamic Balance in the Elderly With Forward Head Posture. *JMR* https://doi.org/10.18502/jmr.v16i1.8556 (2022).

58. Nemmers, T. M., Miller, J. W. & Hartman, M. D. Variability of the Forward Head Posture in Healthy Community-dwelling Older Women:. *J. Geriatr. Phys. Ther.* **32**, 10–14, https://doi.org/10.1519/00139143-200932010-00003 (2009).
